# Divergence in Olfactory Host Plant Preference in *D. mojavensis* in Response to Cactus Host Use

**DOI:** 10.1371/journal.pone.0070027

**Published:** 2013-07-25

**Authors:** Priya Date, Hany K. M. Dweck, Marcus C. Stensmyr, Jodi Shann, Bill S. Hansson, Stephanie M. Rollmann

**Affiliations:** 1 Department of Biological Sciences, University of Cincinnati, Cincinnati, Ohio, United States of America; 2 Department of Evolutionary Neuroethology, Max Planck Institute for Chemical Ecology, Jena, Germany; University of Arkansas, United States of America

## Abstract

Divergence in host adaptive traits has been well studied from an ecological and evolutionary perspective, but identification of the proximate mechanisms underlying such divergence is less well understood. Behavioral preferences for host plants are often mediated by olfaction and shifts in preference may be accompanied by changes in the olfactory system. In this study, we examine the evolution of host plant preferences in cactophilic *Drosophila mojavensis* that feeds and breeds on different cacti throughout its range. We show divergence in electrophysiological responses and olfactory behavior among populations with host plant shifts. Specifically, significant divergence was observed in the Mojave Desert population that specializes on barrel cactus. Differences were observed in electrophysiological responses of the olfactory organs and in behavioral responses to barrel cactus volatiles. Together our results suggest that the peripheral nervous system has changed in response to different ecological environments and that these changes likely contribute to divergence among *D. mojavensis* populations.

## Introduction

Divergence of morphological, physiological, and behavioral traits as a result of local adaptation to different ecological environments is well documented [1]. Studies of host specialization in herbivorous insects, in particular, have been excellent models for understanding adaptive divergence in nature [2], [3]. Conspecific populations can shift to alternate host plants, often because of changes in host plant availability. When such populations are geographically isolated, barriers to gene exchange can further contribute to divergence in host adaptive traits and ultimately may result in reproductive isolation among populations [4], [5].

**Figure 1:**
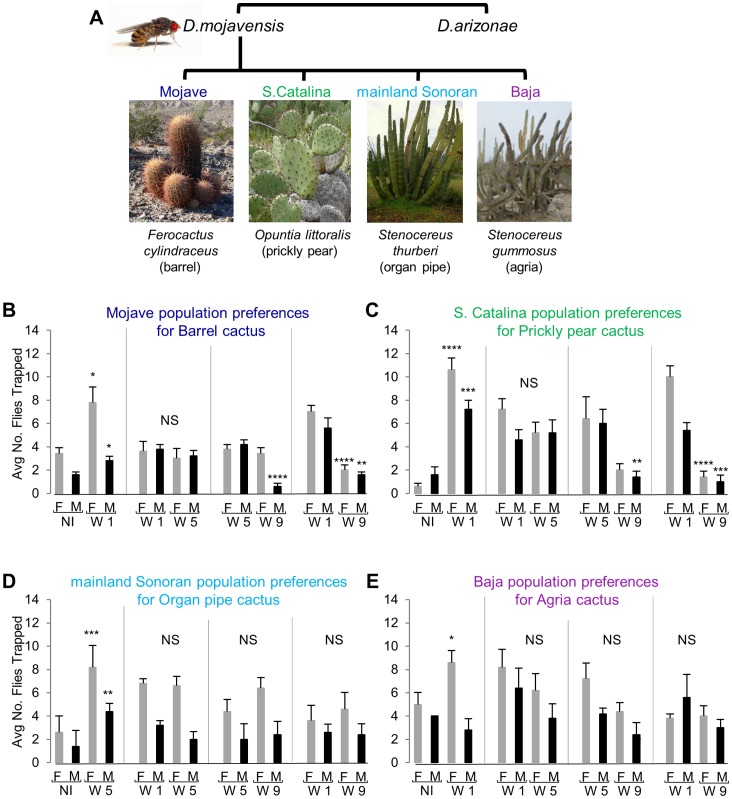
Changes in behavioral preferences with host plant fermentation stage. (**A**) *D. mojavensis* populations specialize on different host cacti across their range. Its sibling species, *D. arizonae* uses columnar cactus and *Opuntia* as hosts. (**B–E**) Two choice behavioral preferences of males (M) and females (F) of the Mojave, S. Catalina, mainland Sonoran, and Baja populations for their own respective host plants. Behavioral preferences for uninoculated (NI) host cactus in comparison to fermented host cactus and preferences for different stages of fermentation of a given host cactus are shown. Cactus tissues were fermented for one to nine weeks (W1– W9). For the mainland Sonoran population, comparisons between uninoculated and five week fermented organ pipe are shown as no significant difference between uninoculated and one week fermented organ pipe was found (data not shown). For panels **B–E**, behavioral preferences are shown as mean ± standard error and significance within a given sex and choice test is depicted by asterisks (*: *P*<0.05; ** *P*<0.01; ***: *P*<0.001; ****: P<0.0001).

Understanding how reproductive isolation evolves requires an examination of the process prior to its completion [3]. Particularly promising are systems in which there is phenotypic divergence among populations of the same species from contrasting environments and for which extensive ecological data have been collected. *Drosophila mojavensis* represents such a system, and thus is a model of incipient speciation. *D. mojavensis* inhabits the arid regions of Baja California and the Sonoran and Mojave deserts of mainland Mexico and southern California and Arizona, respectively [6], [7], [8]. Changes in its range have been accompanied by changes in host plant use, with distinct populations of *D. mojavensis* using different cactus species across its range. The population in Baja California feeds and breeds on pitaya agria (*Stenocereus gummosus*), the mainland Sonoran/Arizona population uses organ pipe cactus (*S. thurberi*) and at times cina (*S. alamosensis*) cactus, and the populations in the Mojave Desert and on Santa Catalina Island utilize barrel (*Ferocactus cylindraceus*) and prickly pear cactus (*Opuntia* spp.), respectively [6], [7] (Figure 1A). The Gulf of California acts as a geographic barrier, restricting gene flow between Baja California and mainland Sonoran populations [9]. These geographically isolated populations show differing levels of premating isolation but no postmating isolation from one another [8], [9], [10], [11]. Its sibling species, *D. arizonae*, ranges from central Guatemala through mainland Mexico to Arizona, using columnar cacti and *Opuntia* hosts [8].

*Drosophila mojavensis* feeds and breeds on necrotic cactus tissue and the volatile compounds produced by the fermenting cactus are the primary sensory cue for host plant identification, and long range attraction to preferred oviposition sites [7], [12]. Early studies of agria and organ pipe rot liquids suggests that host plant chemistry differs between cactus species in the composition and relative amounts of specific compounds [13], [14]. Also, studies of behavioral preferences in *D. mojavensis* for agria and organ pipe rots, or for synthetic mixtures representing the composition of their liquid rots, suggest an overall preference for the agria host [12], [13], [15]. However, knowledge of the volatile compounds that form the odorant headspace surrounding any of the four host cacti is unknown. Moreover, the proximate mechanisms underlying differences in olfactory preferences in this species to host plant volatiles remains to be determined.

Here we examine the evolution of host plant specialization in *D. mojavensis*. We assess the volatile composition of fermenting cactus tissues over time for all four host plants. We then test the hypothesis that adaptation to different host plant volatiles involves alterations at the sensory periphery by examining differences in electrophysiological responses of the olfactory organs among populations. We measure behavioral responses of each population for different fermentation stages of their respective cacti and to specific cactus volatiles. Our findings begin to unravel the mechanisms underlying intraspecific divergence and the evolution of host-plant specialization in *D. mojavensis*.

## Results

### Behavioral Preferences for Cactus Fermentation Stage

The four populations of *D. mojavensis* feed and breed on four different species of fermenting cacti, so we began our study of the role of olfaction in host plant shift by measuring the attraction of each population to their respective host plants across a range of fermentation stages. The purpose of these experiments was to determine at what stage(s) the flies are attracted to their own host cactus necroses, an essential step in identifying the volatile(s) underlying host specific behavioral attraction. Thus, we conducted two choice experiments using a behavioral trap assay system. For each fly population the following comparisons were performed using their own respective host plants: uninoculated (NI) vs. one week (W1) fermented cactus, one week (W1) vs. five week (W5) fermented cactus, five week vs. nine week (W9) fermented cactus and lastly one week vs. nine week fermented cactus. For the mainland Sonoran population, comparisons between uninoculated and five week fermented cacti are shown as no significant difference between uninoculated and one week fermented samples was found (data not shown).

In these behavioral choice tests, all four populations overall showed greater attraction to fermented rather than fresh (uninoculated) cactus tissue (Figure 1B–E). Additionally, preferences in fermentation stage varied among the four populations. The Mojave Desert and S. Catalina populations had clear preferences for earlier fermentation stages (Figure 1B, C; Table S1A, B). Both one week and five week stages were equally attractive to flies, and attraction to nine week old tissue was reduced for both cacti. The mainland Sonoran and Baja populations, on the other hand, were attracted similarly to all of the stages of fermentation (Figure 1D, E; Table S1C, D). Moreover, increased attraction to fermented cactus in the mainland Sonoran population was only observed after five weeks of organ pipe fermentation, despite there being no difference in attractiveness between one and five week fermented samples (Figure 1D). There is also an indication of sex specific responses between some rot stage comparisons and overall females tended to have stronger responses. These results are expected given previous findings in *Drosophila* that show differences in olfactory responses between the sexes and increased behavioral responses in *D. mojavensis* females relative to males [12], [16], [17]. Finally, repetition of these behavioral tests using flies from a second fly line for each population lead to the same conclusion (data not shown), suggesting that changes in attraction with changes in headspace volatiles over time reflect a general population specific result.

### Identification and Comparison of Volatile Composition Over Time

**Figure 2:**
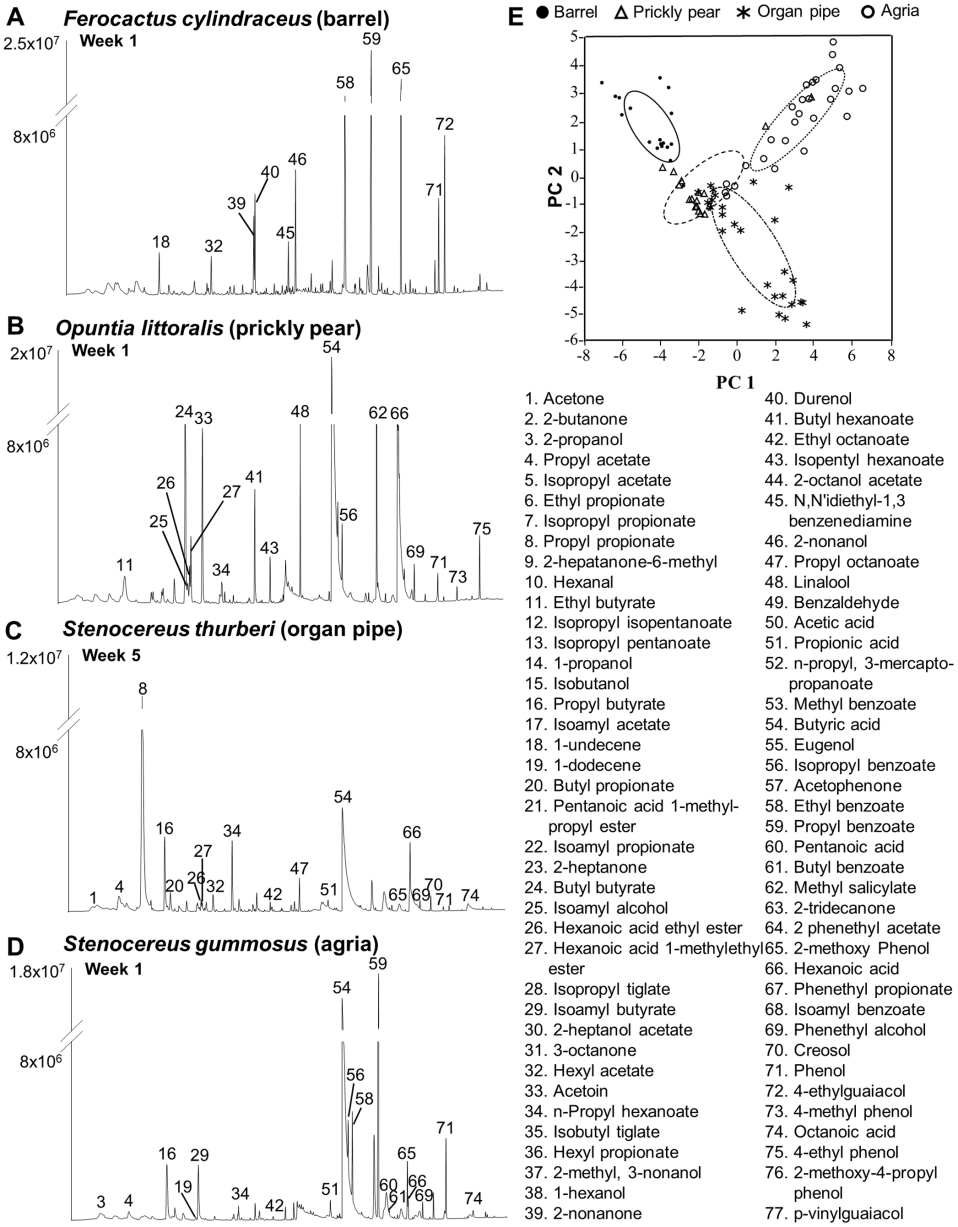
Typical gas chromatograms of headspace for the four cactus hosts. (**A–D**) Barrel, prickly pear, organ pipe and agria cactus headspaces, respectively, from fermented samples are shown. Peak numbers correspond to compounds identified in time course experiment presented in Figure S1. (**E**) Principal component (PC) analysis of the volatile samples from all four cacti. The eigenvectors for the PCs are provided in Table S3. The fifty percent density eclipses for the cacti are indicated with different line styles.

These differences in preference for different fermentation stages arise because volatile composition of fermenting cactus tissues is dynamic [18]. To determine changes in volatiles over time, we sampled the headspace of all four host cacti at one week intervals for nine weeks. Headspace volatiles varied between host plants and varied in their relative amounts across time (Figure 2A–D; Figure S1A–D). Seventy seven compounds were identified, with six unique to barrel, eight to prickly pear, two to agria and none to organ pipe (Table S2). More specifically, 1-dodecene, 2-methoxy-4-propyl phenol, durenol, isopropyl acetate, isopropyl propionate, and N,N'-diethyl-1,3 benzenediamine were unique to barrel cactus. The compounds 2-methyl-3-nonanol, 2-octanol acetate, ethyl propionate, isobutyl tiglate, isopropyl isopentanoate, isopropyl pentanoate, n-propyl 3-mercapto-propanoate, and pentanoic acid 1-methylpropyl ester were unique to prickly pear, and 6-methyl-2-heptanone and acetic acid were unique to agria. In general, the majority of identified compounds were esters (38%) and aromatics (30%). The volatile blends of prickly pear and agria were primarily equal in number of esters and aromatics, but organ pipe and barrel cacti were enriched for esters and aromatics, respectively. These host specific differences are illustrated by a principal component analysis (PCA) based on the volatile composition, in which the four host plants are clearly segregated into separate groups (Figure 2E; Table S3).

### Electrophysiological Responses to Volatiles

**Figure 3:**
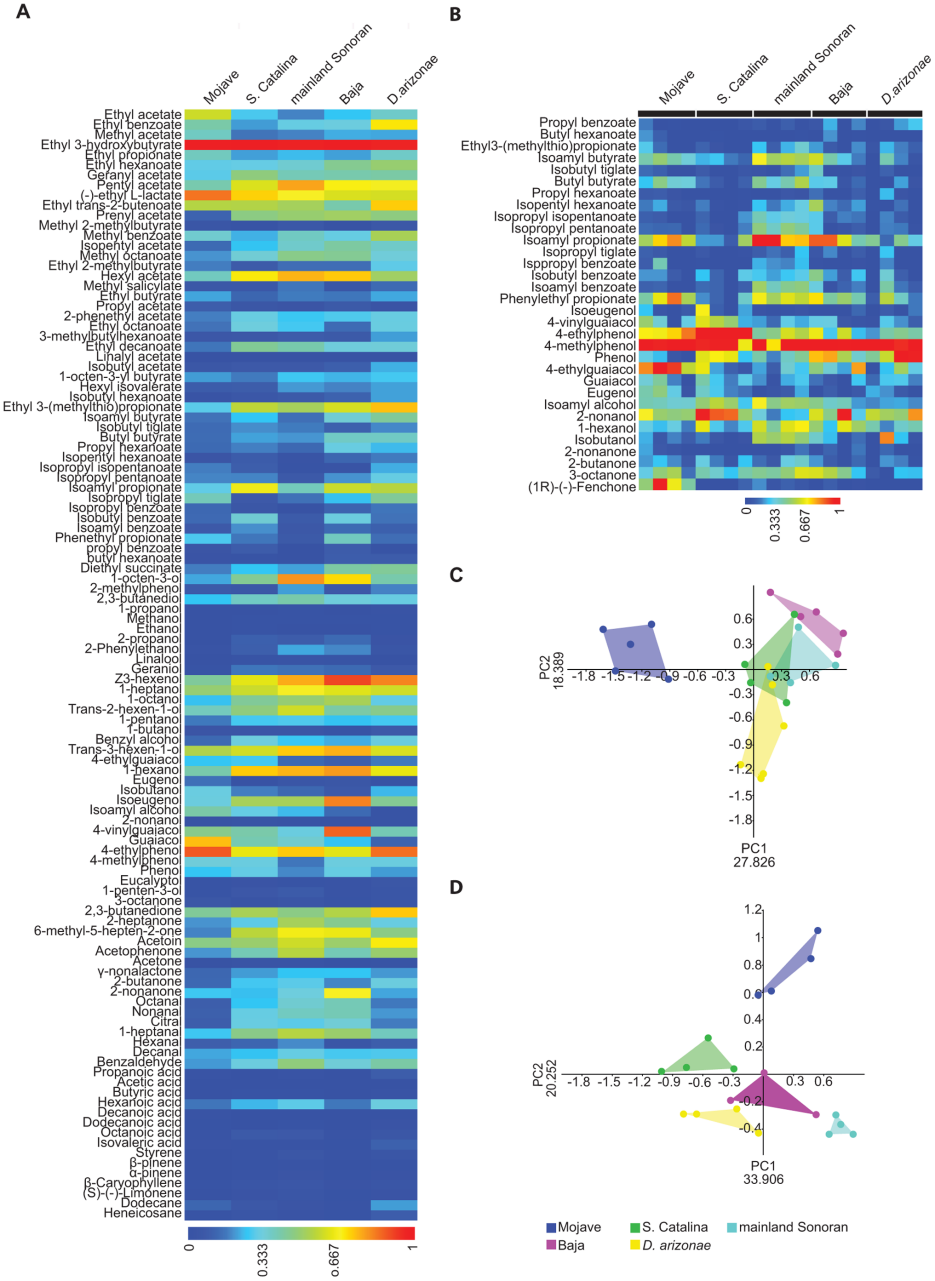
Differences in electrophysiological response properties among the four *D. mojavensis* populations and *D. arizonae*. (**A–B**) Heat map of EAG and EPG responses (respectively) to a suite of odorants for *D. mojavensis* populations and *D. arizonae*. EAG and EPG responses were scaled to a range from 0 to 1. (**C–D**) PCA of EAG and EPG responses, respectively, to a suite of odorants for *D. mojavensis* populations and *D. arizonae*.

Given that the volatile compositions of these host cacti differ substantially, we asked whether there was evidence of host specific adaptations in the olfactory systems of the fly populations. Intraspecific variation in odor-guided behavior has been observed previously in the tephritid fly *Rhagoletis pomonella* and these differences were accompanied by subtle changes in the peripheral odor detection machinery [19], [20]. We examined whether there were alterations in the electrophysiological response properties of the antennae and maxillary palps using electroantennograms (EAG) and electropalpograms (EPG). We measured responses to 110 compounds for the antennae and 32 compounds for the maxillary palps among *D. mojavensis* populations and its sister species, *D. arizonae*. The odorants included diverse chemical groups as well as compounds present in fermenting host cacti ([13], [14] and this study). Both EAG and EPG measurements indicated significant differences in odor detection, especially for the Mojave population (Figure 3A, B), as illustrated by PC analyses based on both EAG and EPG response characteristics. In both cases, the PCA grouped the *D. mojavensis* mainland Sonoran, Baja, and S. Catalina populations together with *D. arizonae*, and separate from the *D. mojavensis* Mojave population (EAG: ANOSIM based on Bray-Curts similarity, R = 0.72, *P*<0.0001, Figure 3C; EPG: ANOSIM based on Bray-Curts similarity, R = 0.625, *P*<0.0001, Figure 3D). Specifically, the antennae of the Mojave population differed primarily in having an overall reduced response to straight-chain esters, and the palps differed in having a strong response to 4-ethylguaiacol, a compound that elicited minimal responses from the other populations and *D. arizonae*. Moreover, as with rot preference behavior, electrophysiological responses did not differ significantly within populations (Table S1E). Therefore, these distinct odor sensitivities of the Mojave population presumably constitute host-specific adaptations. The headspace of barrel cacti, the sole host of the Mojave population, had a lower number of esters and those esters identified typically constitute only minor components of the volatile blend (see section on the identification and comparison of volatile composition over time, above). However, aromatics are a dominant component of barrel cactus headspace, with compounds such as 4-ethylguaiacol, present across all fermentation stages but present only in trace amounts in prickly pear and organ pipe and not detected in agria headspace (Table S2).

### Behavioral Responses to Specific Host Plant Volatiles: Mixtures

**Figure 4:**
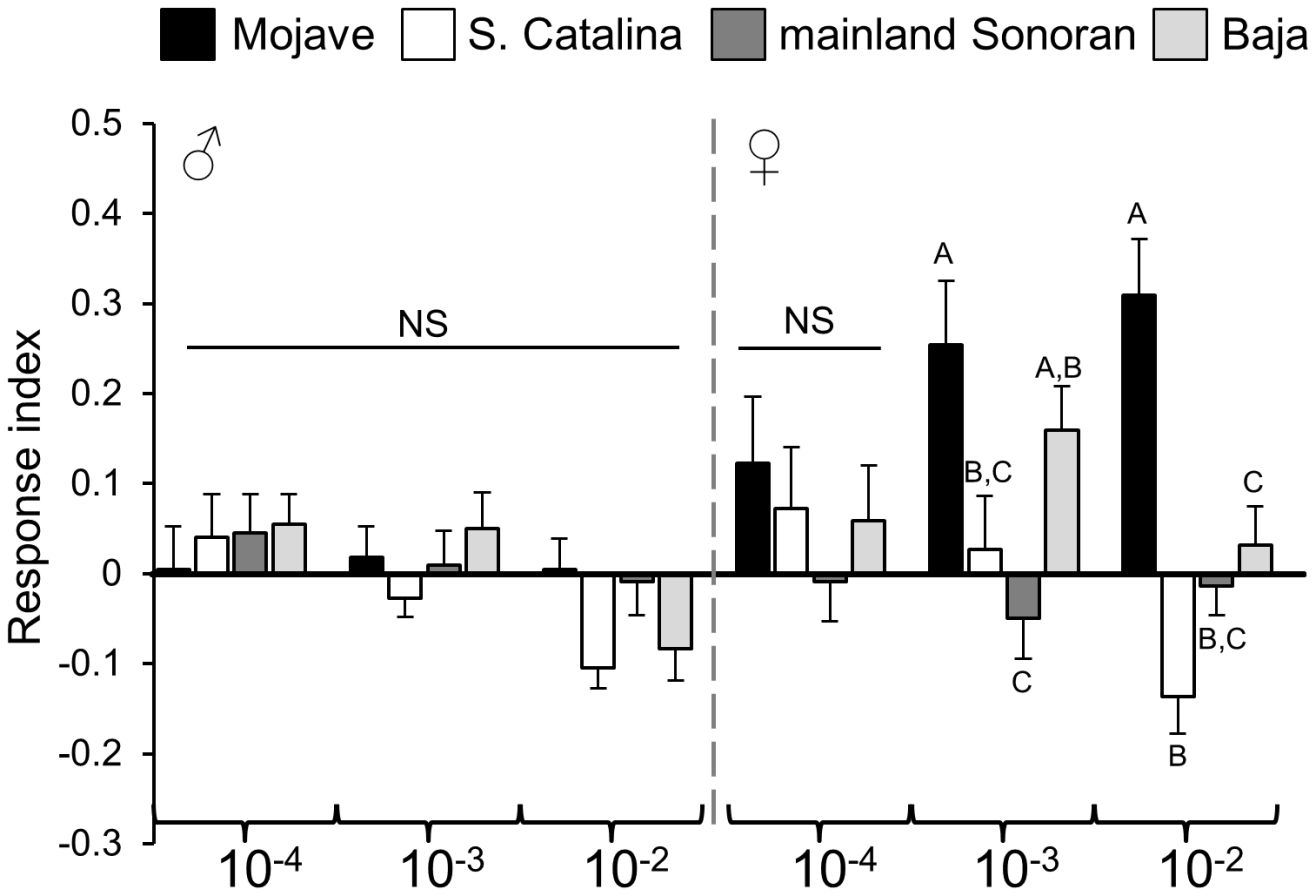
Host specific behavioral preferences for a synthetic mixture of 13 barrel cactus compounds. Behavioral responses of each population to the synthetic mixture were measured at mixture dilutions of 10^−4^, 10^−3^, and 10^−2^. Response indices (mean ± std error) were calculated for each sex and *D. mojavensis* population. Comparisons among populations were made within a given mixture dilution and the letters above the bars denote significant differences in behavioral response among the populations (posthoc Tukey-Kramer test).

**Figure 5:**
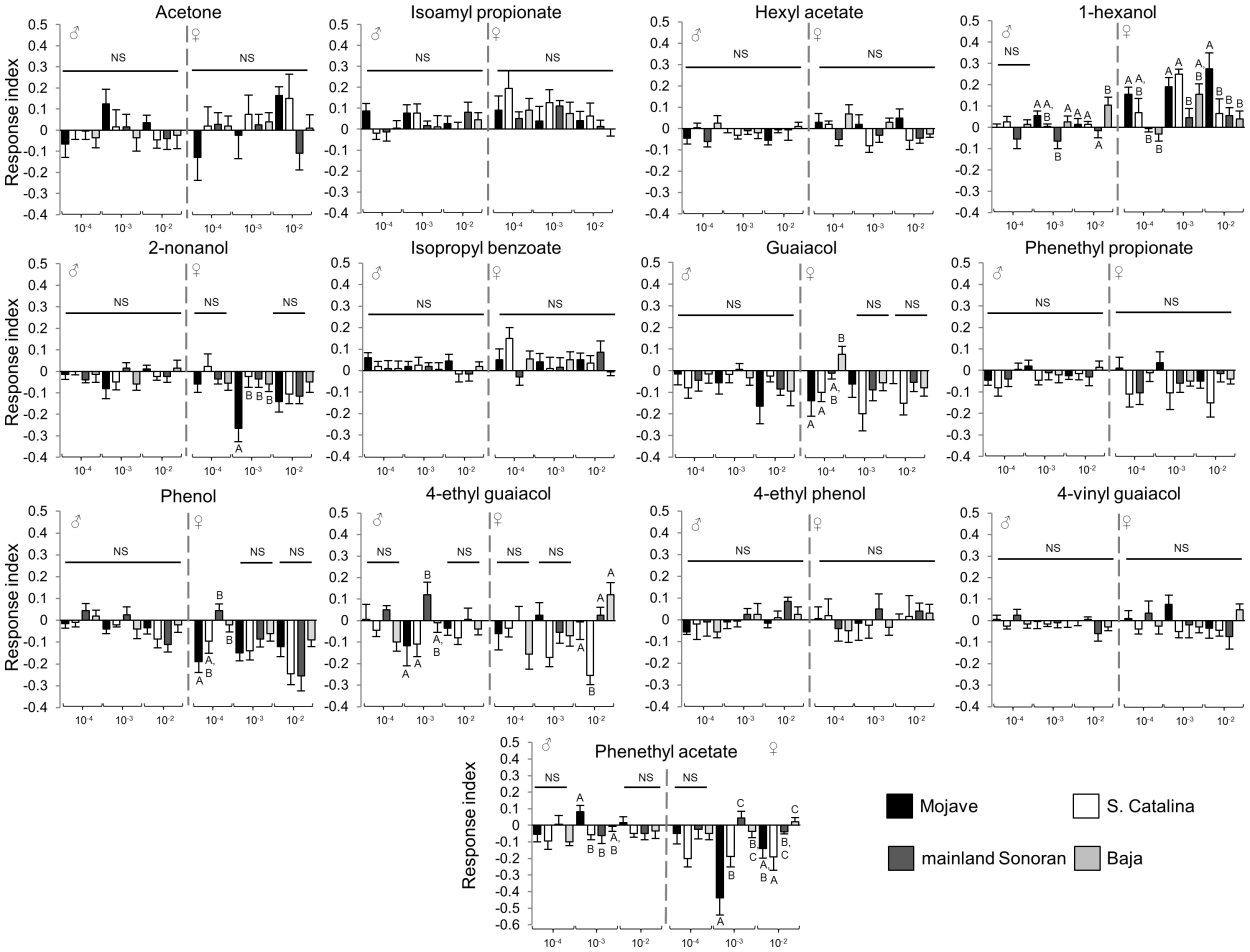
Behavioral responses to single compounds. Dose response indices (mean ± std error) of males (M) and females (F) for single compounds. Significant differences among *D. mojavensis* populations are denoted by different letters above the bars.

Given the shifts in peripheral detection, we measured behavioral responses of the four *D. mojavensis* populations to a mixture of thirteen compounds for which the majority showed population differences in electrophysiological responses and/or were consistently present in barrel cactus. Since responses (electrophysiological and behavioral) did not differ significantly within populations, we focused our behavioral analyses of synthetic compounds on a single line per population. As expected, the Mojave population showed greater attraction to the mixture than the other *D. mojavensis* populations (Figure 4; Table S1F). Females in particular, showed attraction to the mixture in a dose dependent manner, which is consistent with previous studies [16], [17] showing increased behavioral responses in females relative to males. Moreover, females of the Mojave population continued to show attraction to the mixture at a 10^−2^ dilution, while responses of females from the other three populations ranged from decreased attraction to repulsion. In the case of the Baja population, for example, dose responses were shifted to lower concentrations. We also tested responses to the thirteen compounds that were components of the mixture individually, at several concentrations (Figure 5; Table S1G). Most of the single compounds elicited minimal attraction or repulsion across all populations. Furthermore, those single compounds that elicited population specific differences did not recapitulate the host specific responses of the mixture, indicating that a combination of volatiles is essential for appropriate host plant identification and preference.

## Discussion

### Behavioral Responses to Host Plant Volatiles

We observed differences in olfactory preferences in *D. mojavensis* for different stages of host plant fermentation. These fermentation stages varied in the composition and abundance of volatiles produced. Early studies of the Baja population have shown that flies prefer fermenting cactus to fresh tissue and have an attraction to initial fermentation stages [13]. Our results are in accordance with these studies in that the Mojave and Catalina populations also exhibit a preference for early stages of cactus fermentation. No difference in preference among fermentation stages, however, was observed for the mainland Sonoran and Baja populations for their respective host plants. This lack of differential attraction was consistent with comparatively little change in the volatile compositions of these cacti over the test period and most likely reflect methodological differences between our study and Downing, 1985 [13]. Moreover, while rotting in nature likely occurs more rapidly and additional studies on microbe colonization of damaged cactus tissue in nature are needed [21], these laboratory experiments can identify volatiles and changes in volatiles that underlie shifts in host plant preference behavior.

Previous studies of single compounds have been instrumental in the development of our understanding of how olfactory cues are processed [22]. In nature, however, organisms encounter a vast array of volatiles and the importance of single compounds in an ecological context remains less clear. Our results show that individual compounds may elicit behavioral responses in *D. mojavensis*, but that host-specific attraction to a mixture of these compounds could not be explained by responses to a single compound alone. The importance of odor mixtures in mediating appropriate behavioral responses has been observed in other systems (e.g., grapevine moth (*Lobesia botrana*), [23]; oriental fruit moth (*Cydia molesta*), [24]; hawkmoth (*Manduca sexta*) [25]) and our results support a model in which synergistic or antagonistic effects among mixture compounds result in host specific behavioral responses to olfactory cues.

### Determinants of Olfactory Preferences

Adaptation to different ecological environments can result in divergence of olfactory preference [26]. We examined whether alterations at the sensory periphery were found among populations of *D. mojavensis* that differ in their host plant use. Notably we observed that the Mojave Desert population, specializing on barrel cactus, has diverged in its olfactory sensitivities with an overall decreased response to esters and increased response to aromatics. This divergence from the other three populations coincides with the fact that the volatiles released by fermenting barrel cactus are heavy in aromatics compared to the other three host cacti whose primary volatiles were enriched for esters or balanced equally with aromatics ([13], [14] and this study). These electrophysiological differences most likely reflect alterations in ligand binding or odorant clearance at the sensory periphery, either through changes in gene expression or protein structure-function. The latter, caused by amino acid substitutions in chemosensory receptors, has been shown to confer differences in odorant sensitivity [27], [28], [29]. On the other hand, changes in the number of olfactory sensory neurons can also tune olfactory sensitivity towards host-specific volatiles, as in the case of *D. sechellia*, a specialist on *Morinda* fruit [30], [31]. Moreover, this preference by *D. sechellia* for *Morinda* fruit volatiles has been shown to be mediated by odorant binding proteins [32] and host-driven sensory augmentation has been shown for other insects, such as *Culex* mosquitoes [33].

### The Evolution of Olfactory Preference in *D. mojavensis*

In short, we have begun to understand the evolution of olfactory preference in response to host plant shift in *D. mojavensis*, a model of incipient speciation. Our results suggest rapid adaptation to changes in host plant utilization in this system. Estimates of divergence between.

*D. mojavensis* and *D. arizonae* range between 1.91 and 2.97 million years ago [34], [35]. Moreover, Smith et al., 2012 [36] estimates that the Baja population diverged from an ancestral mainland Sonoran/Mojave Desert group 230,000 to 270,000 years ago. Separation of the mainland Sonoran and Mojave Desert populations are then estimated to have occurred 117,000 to 135,000 years ago. Such rapid adaptation of the olfactory system has also been observed in *Rhagoletis*, with shifts in olfactory preferences from hawthorn to apple within 150 years [37]. In the aforementioned studies of shift of host preference in *D. sechellia*, the shift was proposed to be in response to competition with *D. simulans* [38], with divergence between species less than half a million years ago [39]. Our findings in this system will help unravel mechanisms underlying the process of species formation and the evolution host-plant specialization.

## Materials and Methods

### Identification of Host Plant Volatiles

An analysis of the volatile compositions of *Stenocereus gummosus*, *S. thurberi*, *Ferocactus cylindraceus*, and *Opuntia littoralis*, was obtained through headspace solid phase microextraction (SPME, Polydimethylsiloxane/Divinylbenzene, Sigma-Aldrich, St. Louis, MO). For each cactus, volatile compounds emitted from uninoculated and inoculated cactus were identified. The tissue was kept frozen and for the experiments it was thawed, placed in a glass jar with a polyethylene lined cap, sterilized and subsequently inoculated. More specifically, a 70 g piece of cactus tissue was inoculated with 1.0 ml of seven yeast species (*Pichia cactophila, P. mexicana, Starmera amethionina, Candida valida, C. sonorensis, Diapodascus starmeri and Sporopachyderma cereana*) mixture and 0.5 ml of one pectolytic bacterium *Erwinia cacticida* ([40], [41] and Etges pers. comm.). Both yeast and bacteria cultures were freshly grown on Yeast Complete media or Glucose Yeast Calcium carbonate media plates respectively. After 48 hours, the microorganisms were harvested and suspended in sterile water. To facilitate the even distribution of the microorganisms, the cactus tissue was subsequently inoculated at multiple spots using a syringe [40], [41]. These yeast species have been documented on necrotic cacti [18] and used previously in *D. mojavensis* rearing experiments [41]. Cactus tissue was then incubated at 30°C and the volatiles present in the headspace were determined at weekly intervals over nine weeks. The volatile compositions of two to three replicate rots per cactus were examined over the nine week period. The identification of volatiles emitted from uninoculated cactus tissue was determined after one day.

The SPME fiber was exposed for one hour to the sample headspace, and the fiber assembly was then placed into the GC-MS injector port. Volatiles were analyzed using an Agilent 7890A GC with 5975C MSD apparatus (Santa Clara, CA) in a pulsed splitless mode. The GC-MS was equipped with a polyethylene glycol column (Nukol, Supelco Co.). GC conditions were optimized with standards and subsequent analyses done at injector and detector (FID) temperatures of 250°C and 280°C, respectively. Helium was used as the carrier gas at 25 ml min^−1^, and at a split ratio of 2∶1. The oven temperature was initially set at 40°C for 1 min and then ramped to 210°C at a rate of 7° min^−1^. Mass spectra were recorded from 35 to 700 amu, with electronic impact ionization at 70eV. Compounds were identified using the NIST Mass Spectral Library, by comparison to their retention times, and by mass spectra analyses of select standards. Compounds with more than a 90% match with the NIST library were labeled. Raw data was subjected to principal component analysis (PCA) using JMP 9.0 (SAS Institute, Cary, NC).

### *Drosophila* Stocks

Flies were obtained from the Drosophila Species Stock Center or kindly provided by Dr. Bill Etges and are as follows: Baja California population, [Punta Prieta (stock number 15081–1351.30) and San Quintin (SQ59a)]; the mainland Sonoran population [Organ Pipe National Monument, Arizona, stock number 15081–1352.32 and OPNM9]; the Mojave population [Grand Canyon, Arizona (stock number 15081–1352.10) and Providence Mountain, CA (A997b)]; Santa Catalina Island [stock numbers 15081–1352.30 and 15081–1352.22]; *D. arizonae* [Sinaloa, Mexico (stock number 15081–1271.33)]. All flies were reared on cactus-banana-agar medium and were maintained at 25 ^o^C, under a 12 h L/D cycle.

### Electrophysiological Recordings

#### Odorants

Pure odorants were diluted (10^−3^) in hexane or in water as appropriate. Diluted odors (10 µl) were pipetteted onto a small piece of filter paper (∼1 cm^2^) and placed inside a glass Pasteur pipette. For odorant application, a stimulus controller was used (Stimulus Controller CS-55, Syntech, Hilversum, The Netherlands). All odorants were obtained from Sigma-Aldrich (St. Louis, MO) at the highest purity available.

#### Electroantennograms/electropalpograms

Individual flies were immobilized in a pipette tip with the head partially protruding. Reference and recording glass capillary electrodes were filled with haemolymph Ringers. The reference electrode was inserted into one eye and the recording electrode brought into contact with either the proximal third antennal segment or the distal portion of the maxillary palps. A constant flow of charcoal-filtered and humidified air (1 l min^−1^) was delivered at a velocity of 0.5 ms^−1^, through a tube with its outlet approximately 10 mm from the antenna/palp. Odorant was introduced by placing the tip of the pipette through a hole in the side of this tube. The EAG signal (transferred via Ag-AgCl wires) was pre-amplified (10x) with a probe connected to a high-impedance DC-amplifier (EAG-probe Version2, Syntech) and digitally converted (IDAC-4 USB, Syntech), visualized and recorded on a PC using a dedicated software (EAG-probe, Syntech). Recordings were obtained from 2–4 individuals per sex and line. Traces of individual flies were scaled to a range from 0 to 1. Quantitative reactions to odor compounds were used for principal component analysis (via variance covariance). Calculations were done with PAST (http://folk.uio.no/ohammer/past/download.html) and SPSS software Version 17 (SPSS, www. Spss.com). To assess the degree of similarity between lines within a population, electrophysiological responses were measured to eleven odorants for lines of the Mojave population and the S. Catalina population. The odorants were selected based on their ability to elicit a range of responses.

### Behavioral Trap Assay

Free walking behavioral assays consisted of twenty flies placed into a polystyrene arena (6 cm (H) ×15 cm (Ø)) containing two traps. Each trap was constructed using a 10 ml glass beaker, fitted with a polypropylene plastic funnel. Traps were then symmetrically placed within the testing arena. To prevent dehydration of the flies, a cotton ball saturated with 20 ml of water was placed into the arena. Flies were tested at 10–12 days post-eclosion and flies were starved overnight prior to the experiment. Assays were performed in the dark and the number of flies trapped was recorded after 48 hours. For tests of single odorants or synthetic mixtures, traps contained 2 ml of the vehicle control and 0.1% Triton X with or without odorant(s). All odorants were obtained from Sigma-Aldrich (St. Louis, MO) at the highest purity available. Behavioral responses were measured to a synthetic mixture of 13 individual compounds at proportions reflective of the headspace of a three week fermented barrel cactus (Table S2). These mixture included hexyl acetate, acetone, phenethyl acetate, guaiacol, 1-hexanol, 2-nonanol, 4-ethyl guaiacol, phenol, isopropyl benzoate, phenethyl propionate, isoamyl propionate, 4-ethyl phenol, and 4-vinyl guaiacol. Because 4-vinyl guaiacol was present in the majority of early stage rots, with the exception of week 3, it was included at its average relative amount across weeks one through five. All mixture components were also tested singly. Response indices were calculated by subtracting the number of flies present in the control traps from the number of flies present in the trap containing odor and dividing by the total number of flies. Ten replicate measurements per sex, population and odorant concentration were conducted. Statistical analyses were conducted using ANOVA, followed by a Tukey-Kramer post-hoc test. For behavioral tests using fermenting cactus, all four cacti were inoculated as described in the above identification of host plant volatile section. Two grams of uninoculated or fermented cactus tissue was used per trap. Five replicate measurements per sex and per choice test were conducted. Statistical analyses were conducted within a given sex and two choice test using ANOVA. All analyses were done using JMP 9.0 software (SAS Institute, Cary, NC).

## Supporting Information

Figure S1 Analysis of host plant volatile composition with cactus rot stage. Cacti were either uninoculated (NI) or inoculated and fermented for one to nine weeks. Peak numbers correspond to the list of volatiles. **(A–D)** Typical gas chromatograms of barrel, prickly pear, organ pipe and agria headspace (respectively) from uninoculated or representative fermented samples (weeks 1, 5, and 9).
(PDF)

Table S1 Analysis of variance for all experiments. **(A–D)** Experiments testing preference for different fermentation stages of barrel, prickly pear, organ pipe and agria cacti, respectively. **(E)** Comparisons of electrophysiological responses between lines within a *D. mojavensis* population. **(F)** Behavioral responses to the synthetic mixture. **(G)** Behavioral responses of single compounds.
(PDF)

Table S2 Relative amounts of volatile compounds in uninoculated and inoculated cacti. Volatile compounds (mean ± stdev) emitted from barrel, prickly pear, organ pipe and agria cacti.
(PDF)

Table S3 Principal component values for volatile compounds in the four host cacti. Eigenvectors with highest scores are indicated in bold. The compounds which were present only once across all four cacti were excluded from the PCA.
(PDF)
