# Correction: Divergence in Olfactory Host Plant Preference in *D. mojavensis* in Response to Cactus Host Use

**DOI:** 10.1371/journal.pone.0090050

**Published:** 2014-02-18

**Authors:** The PLOS ONE Staff

Panels A and B in Supplementary Figure S1 are switched. The panel labels are not switched. Please see a corrected version of Supplementary Figure S1 below.

## Supporting Information

Figure S1 **Analysis of host plant volatile composition with cactus rot stage.** Cacti were either uninoculated (NI) or inoculated and fermented for one to nine weeks. Peak numbers correspond to the list of volatiles. (A–D) Typical gas chromatograms of barrel, prickly pear, organ pipe and agria headspace (respectively) from uninoculated or representative fermented samples (weeks 1, 5, and 9).
(PDF)
