# Supplementary material for: Divergence in Olfactory Host Plant Preference in D. mojavensis in Response to Cactus Host Use
Source: PLoS One. 2013 Jul 25;8(7):e70027. doi: 10.1371/journal.pone.0070027 (PMC3723661; doi:10.1371/journal.pone.0070027)
Supplement: Figure S1 — Analysis of host plant volatile composition with cactus rot stage. Cacti were either uninoculated (NI) or inoculated and fermented for one to nine weeks. Peak numbers correspond to the list of volatiles. (A–D) Typical gas chromatograms of barrel, prickly pear, organ pipe and agria headspace (respectively) from uninoculated or representative fermented samples (weeks 1, 5, and 9). (PDF) [file pone.0070027.s001.pdf]

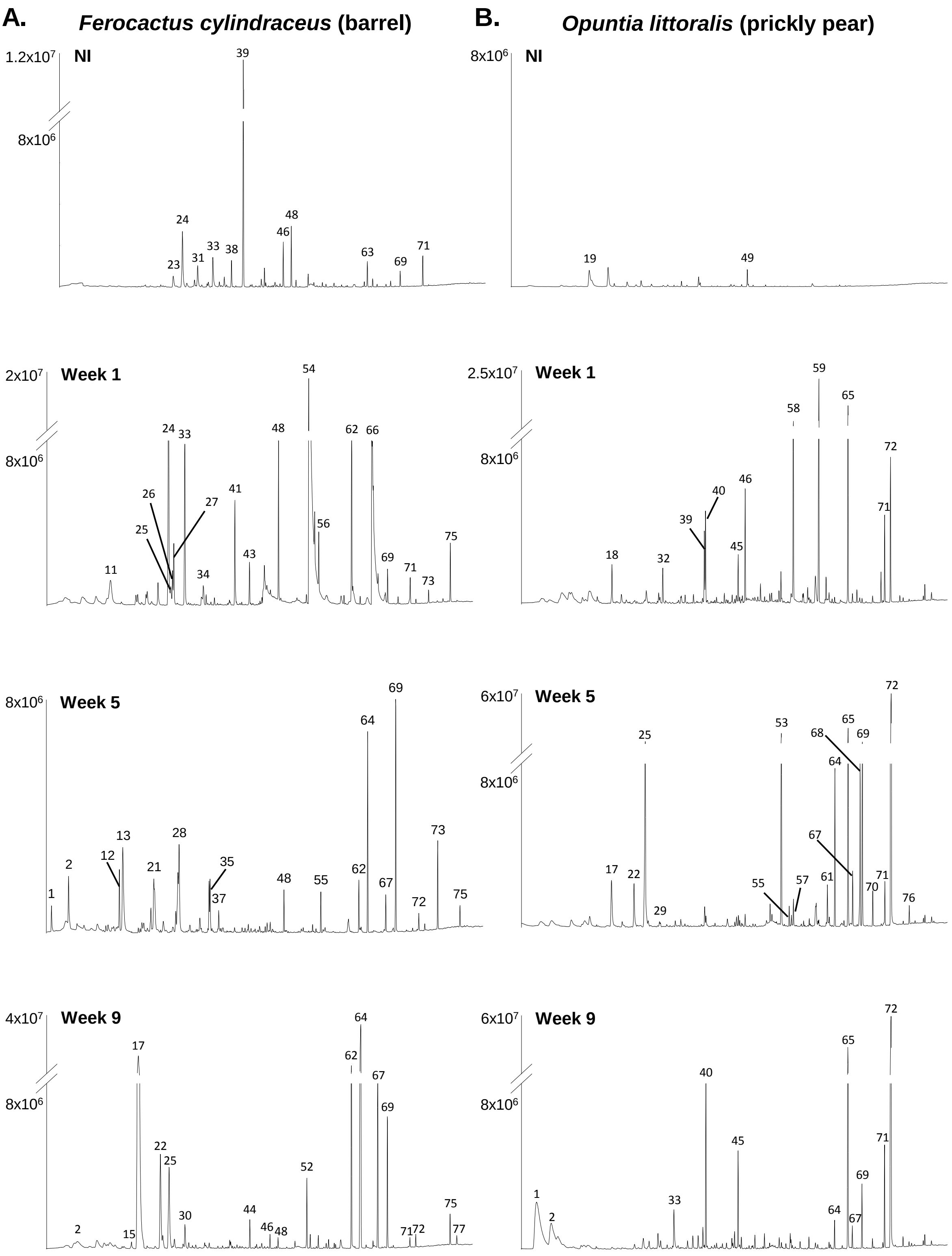

A.
Ferocactus cylindraceus (barrel)
B.
Opuntia littoralis (prickly pear)
1.2x10^7
8x10^6
NI
39
24
48
46
23
31
33
38
63
69
71
8x10^6
NI
19
49
2x10^7
8x10^6
Week 1
54
24
33
48
62
66
26
27
25
41
56
11
43
34
69
71
73
75
2.5x10^7
8x10^6
Week 1
59
58
65
72
40
46
39
71
18
32
45
8x10^6
Week 5
69
64
13
12
2
28
21
35
37
1
48
55
62
67
72
73
75
6x10^7
8x10^6
Week 5
72
25
53
65
68
69
64
67
17
22
29
55
57
61
70
71
76
4x10^7
8x10^6
Week 9
17
64
62
67
69
22
25
52
2
15
30
44
46
48
71
72
75
77
6x10^7
8x10^6
Week 9
72
65
40
45
71
69
1
2
33
64
67

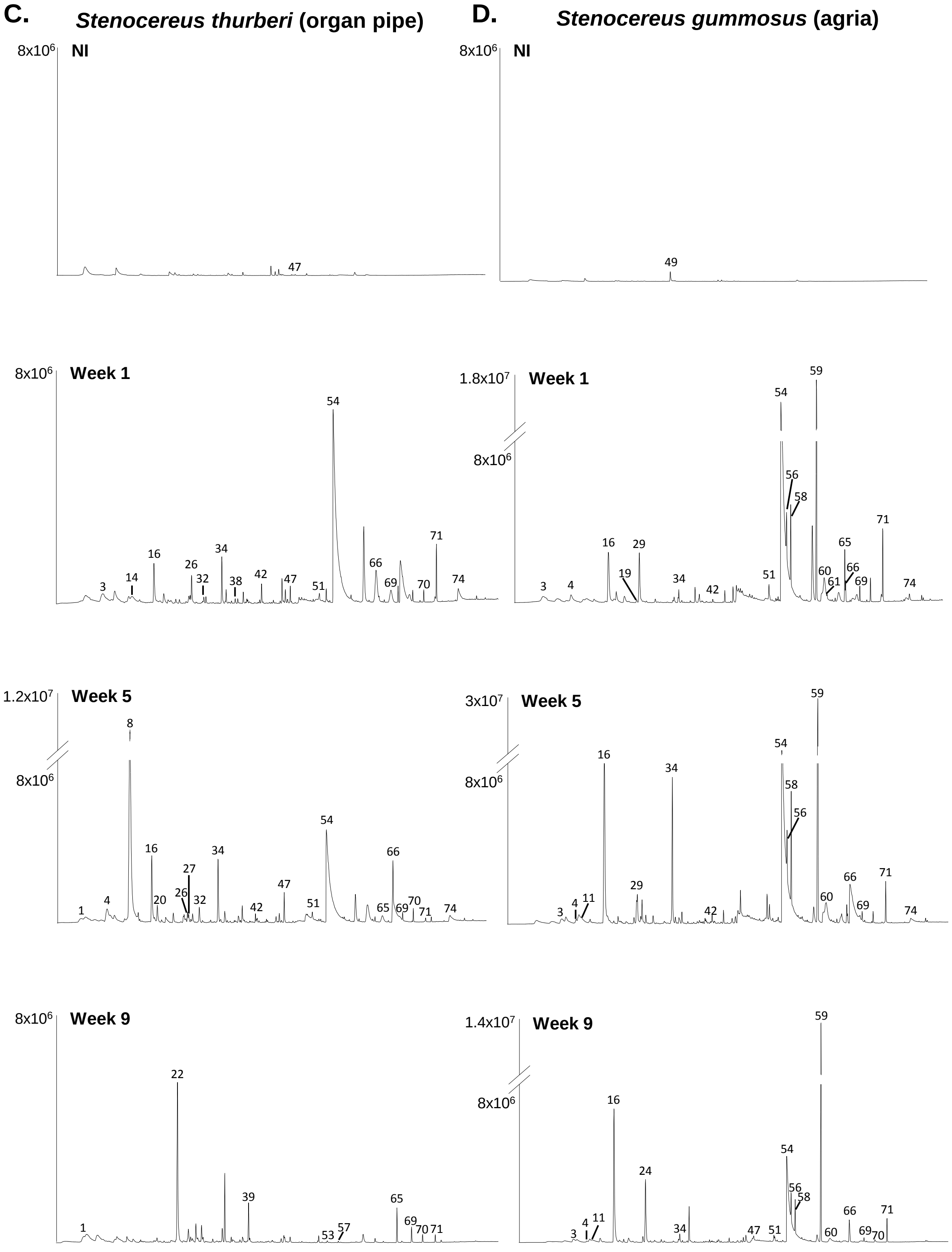
C.
Stenocereus thurberi (organ pipe)
D.
Stenocereus gummosus (agria)
8x10^6
NI
47
8x10^6
NI
49
8x10^6
Week 1
3 14 16 26 32 34 38 42 47 51 54 66 69 70 71 74
1.8x10^7
8x10^6
Week 1
3 4 16 19 29 34 42 51 54 56 58 59 60 61 65 66 69 71 74
1.2x10^7
8x10^6
Week 5
1 4 8 16 20 26 27 32 34 42 47 51 54 65 66 69 70 71 74
3x10^7
8x10^6
Week 5
3 4 11 16 29 34 42 54 56 58 59 60 66 69 71 74
8x10^6
Week 9
1 22 39 53 57 65 69 70 71
1.4x10^7
8x10^6
Week 9
3 4 11 16 24 34 47 51 54 56 58 59 60 66 69 70 71
