# Supplementary material for: Divergence in Olfactory Host Plant Preference in D. mojavensis in Response to Cactus Host Use
Source: PLoS One. 2013 Jul 25;8(7):e70027. doi: 10.1371/journal.pone.0070027 (PMC3723661; doi:10.1371/journal.pone.0070027)
Supplement: Table S1 — Analysis of variance for all experiments. (A–D) Experiments testing preference for different fermentation stages of barrel, prickly pear, organ pipe and agria cacti, respectively. (E) Comparisons of electrophysiological responses between lines within a D. mojavensis population. (F) Behavioral responses to the synthetic mixture. (G) Behavioral responses of single compounds. (PDF) [file pone.0070027.s002.pdf]

Table S1. Analysis of variance for all experiments.

A. Barrel fermentation	Analysis	Source of Variation	d.f.	Mean squares	F ratio	p-value
Uninoculated vs. Fermentation week 1	Females	Line	1	48.400	9.68	0.0140
		Error	8	5.000		
	Males	Line	1	3.600	7.20	0.0270
		Error	8	0.500		
Fermentation week 1 vs. week 5	Females	Line	1	0.900	0.25	0.6320
		Error	8	3.650		
	Males	Line	1	0.900	0.95	0.3580
		Error	8	0.950		
Fermentation week 5 vs. week 9	Females	Line	1	0.400	0.40	0.5440
		Error	8	1.000		
	Males	Line	1	32.400	64.80	0.0001
		Error	8	0.500		
Fermentation week 1 vs. week 9	Females	Line	1	62.500	50.00	0.0001
		Error	8	1.250		
	Males	Line	1	40.000	19.51	0.0020
		Error	8	2.050		
**B. Prickly pear fermentation**	**Analysis**	**Source of Variation**	**d.f.**	**Mean squares**	**F ratio**	**p-value**
Uninoculated vs. Fermentation week 1	Females	Line	1	250.000	89.29	0.0001
		Error	8	2.800		
	Males	Line	1	78.400	28.51	0.0007
		Error	8	2.750		
Fermentation week 1 vs. week 5	Females	Line	1	10.000	2.32	0.1610
		Error	8	4.200		
	Males	Line	1	0.900	0.18	0.6820
		Error	8	5.000		
Fermentation week 5 vs. week 9	Females	Line	1	48.400	5.02	0.0500
		Error	8	9.650		
	Males	Line	1	52.900	12.03	0.0085
		Error	8	4.400		
Fermentation week 1 vs. week 9	Females	Line	1	184.900	63.76	0.0001
		Error	8	2.900		
	Males	Line	1	48.400	25.47	0.0010
		Error	8	1.900		
**C. Organ pipe fermentation**	**Analysis**	**Source of Variation**	**d.f.**	**Mean squares**	**F ratio**	**p-value**
Uninoculated vs. Fermentation week 5	Females	Line	1	78.400	39.20	0.0002

		Error	8	2.000		
	Males	Line	1	22.500	14.51	0.0052
		Error	8	1.550		
Fermentation week 1 vs. week 5	Females	Line	1	0.100	0.02	0.8882
		Error	8	4.700		
	Males	Line	1	3.600	0.45	0.5174
		Error	8	7.800		
Fermentation week 5 vs. week 9	Females	Line	1	10.000	1.04	0.3361
		Error	8	9.550		
	Males	Line	1	0.400	0.11	0.7404
		Error	8	3.400		
Fermentation week 1 vs. week 9	Females	Line	1	2.500	0.23	0.6395
		Error	8	10.550		
	Males	Line	1	0.100	0.02	0.8709
		Error	8	3.550		
**D. Agria fermentation**	**Analysis**	**Source of Variation**	**d.f.**	**Mean squares**	**F ratio**	**p-value**
Uninoculated vs. Fermentation week 1	Females	Line	1	32.400	6.00	0.0400
		Error	8	5.400		
	Males	Line	1	3.600	1.53	0.2509
		Error	8	2.350		
Fermentation week 1 vs. week 5	Females	Line	1	10.000	0.89	0.3724
		Error	8	11.200		
	Males	Line	1	16.900	1.50	0.2552
		Error	8	11.250		
Fermentation week 5 vs. week 9	Females	Line	1	19.600	3.26	0.1083
		Error	8	6.600		
	Males	Line	1	8.100	2.49	0.1531
		Error	8	3.250		
Fermentation week 1 vs. week 9	Females	Line	1	0.100	0.04	0.8417
		Error	8	2.350		
	Males	Line	1	16.900	1.55	0.2483
		Error	8	10.900		
**E. Electrophysiological responses between line**	**Analysis**	**Source of Variation**	**d.f.**	**Sum of squares**	**F ratio**	**p-value**
	Mojave	Line	1	0.220	0.23	0.6328
		Odor	10	36.520	3.75	0.0007
		Line X Odor	10	8.540	0.88	0.5599
	S Catalina	Line	1	1.287	1.91	0.1730
		Odor	10	61.564	9.12	0.0001
		Line X Odor	10	4.689	0.69	0.7253

F. Behavioral responses to the Synthetic mixture	Analysis	Source of Variation	d.f.	Mean squares	F ratio	p-value
10^{-2} Dilution	Females	Line	3	0.389	16.55	0.0001
		Error	40	0.023		
	Males	Line	3	0.031	2.73	0.0575
		Error	38	0.011		
10^{-3} Dilution	Females	Line	3	0.202	5.68	0.0020
		Error	40	0.035		
	Males	Line	3	0.011	0.86	0.4690
		Error	40	0.012		
10^{-4} Dilution	Females	Line	3	0.032	0.76	0.5240
		Error	40	0.042		
	Males	Line	3	0.005	0.26	0.8520
		Error	40	0.020		

G. Behavioral responses to the Single compounds	Analysis	Source of Variation	d.f.	Mean squares	F ratio	p-value
**Acetone**						
10^{-2} Dilution	Females	Line	3	0.167	2.67	0.0620
		Error	36	0.062		
	Males	Line	3	0.013	0.57	0.6380
		Error	36	0.023		
10^{-3} Dilution	Females	Line	3	0.017	0.28	0.8400
		Error	36	0.061		
	Males	Line	3	0.045	0.95	0.4250
		Error	36	0.047		
10^{-4} Dilution	Females	Line	3	0.057	0.92	0.4420
		Error	35	0.062		
	Males	Line	3	0.009	0.37	0.7760
		Error	36	0.024		
**Isoamyl propionate**						
10^{-2} Dilution	Females	Line	3	0.009	0.42	0.7380
		Error	40	0.021		
	Males	Line	3	0.012	0.82	0.4890
		Error	40	0.015		
10^{-3} Dilution	Females	Line	3	0.014	0.53	0.6619
		Error	31	0.026		
	Males	Line	3	0.012	0.81	0.4970
		Error	32	0.015		
10^{-4} Dilution	Females	Line	3	0.042	0.97	0.4170
		Error	40	0.043		
	Males	Line	3	0.026	1.71	0.1790
		Error	40	0.015		
**Hexyl acetate**						

10^{-2} Dilution	Females	Line	3	0.020	2.08	0.1202
		Error	36	0.010		
	Males	Line	3	0.008	0.90	0.4483
		Error	36	0.008		
10^{-3} Dilution	Females	Line	3	0.020	2.23	0.1012
		Error	36	0.010		
	Males	Line	3	0.001	0.37	0.7732
		Error	36	0.004		
10^{-4} Dilution	Females	Line	3	0.020	2.20	0.1051
		Error	36	0.010		
	Males	Line	3	0.010	2.09	0.1189
		Error	36	0.007		
**1-hexanol**						
10^{-2} Dilution	Females	Line	3	0.120	3.85	0.0173
		Error	36	0.030		
	Males	Line	3	0.020	3.22	0.0325
		Error	36	0.008		
10^{-3} Dilution	Females	Line	3	0.070	4.32	0.0106
		Error	36	0.010		
	Males	Line	3	0.026	3.88	0.0167
		Error	36	0.006		
10^{-4} Dilution	Females	Line	3	0.060	4.09	0.0135
		Error	36	0.010		
	Males	Line	3	0.010	1.53	0.2224
		Error	36	0.008		
**2-nonanol**						
10^{-2} Dilution	Females	Line	3	0.010	0.71	0.5498
		Error	36	0.020		
	Males	Line	3	0.004	0.69	0.5641
		Error	36	0.006		
10^{-3} Dilution	Females	Line	3	0.120	5.61	0.0029
		Error	36	0.020		
	Males	Line	3	0.010	1.40	0.2593
		Error	36	0.010		
10^{-4} Dilution	Females	Line	3	0.010	0.87	0.4666
		Error	35	0.010		
	Males	Line	3	0.002	0.48	0.7010
		Error	35	0.005		
**Isopropyl benzoate**						
10^{-2} Dilution	Females	Line	3	0.010	1.03	0.3928
		Error	36	0.010		
	Males	Line	3	0.008	0.91	0.4458
		Error	36	0.009		

10^{-3} Dilution	Females	Line	3	0.003	0.19	0.9000
		Error	36	0.010		
	Males	Line	3	0.001	0.09	0.9635
		Error	36	0.008		
10^{-4} Dilution	Females	Line	3	0.050	2.74	0.0576
		Error	36	0.010		
	Males	Line	3	0.005	0.61	0.6112
		Error	36	0.009		
**Guaiacol**						
10^{-2} Dilution	Females	Line	3	0.038	1.55	0.2170
		Error	36	0.024		
	Males	Line	3	0.032	1.03	0.3920
		Error	36	0.032		
10^{-3} Dilution	Females	Line	3	0.040	1.28	0.2970
		Error	32	0.031		
	Males	Line	3	0.006	0.43	0.7330
		Error	32	0.014		
10^{-4} Dilution	Females	Line	3	0.091	3.99	0.0152
		Error	35	0.022		
	Males	Line	3	0.009	0.42	0.7430
		Error	36	0.023		
**Phenethyl propionate**						
10^{-2} Dilution	Females	Line	3	0.030	1.87	0.1526
		Error	36	0.010		
	Males	Line	3	0.004	0.47	0.7118
		Error	36	0.008		
10^{-3} Dilution	Females	Line	3	0.034	1.25	0.3050
		Error	36	0.020		
	Males	Line	3	0.007	0.75	0.5310
		Error	36	0.009		
10^{-4} Dilution	Females	Line	3	0.030	1.45	0.2446
		Error	36	0.020		
	Males	Line	3	0.010	1.14	0.3464
		Error	36	0.010		
**Phenol**						
10^{-2} Dilution	Females	Line	3	0.070	2.86	0.0506
		Error	36	0.020		
	Males	Line	3	0.010	1.48	0.2352
		Error	36	0.010		
10^{-3} Dilution	Females	Line	3	0.010	1.34	0.2767
		Error	36	0.010		
	Males	Line	3	0.009	0.98	0.4100
		Error	36	0.009		

10^{-4} Dilution	Females	Line	3	0.100	5.49	0.0033
		Error	36	0.010		
	Males	Line	3	0.007	1.22	0.3190
		Error	36	0.006		
**4-ethylguaiacol**						
10^{-2} Dilution	Females	Line	3	0.255	8.47	0.0002
		Error	35	0.030		
	Males	Line	3	0.012	0.88	0.4590
		Error	36	0.013		
10^{-3} Dilution	Females	Line	3	0.061	2.48	0.0775
		Error	35	0.025		
	Males	Line	3	0.120	3.01	0.0429
		Error	35	0.039		
10^{-4} Dilution	Females	Line	3	0.042	1.04	0.3870
		Error	35	0.041		
	Males	Line	3	0.041	2.09	0.1180
		Error	36	0.019		
**4-ethyl phenol**						
10^{-2} Dilution	Females	Line	3	0.003	0.10	0.9623
		Error	36	0.030		
	Males	Line	3	0.010	2.40	0.0837
		Error	36	0.010		
10^{-3} Dilution	Females	Line	3	0.010	0.36	0.7776
		Error	36	0.040		
	Males	Line	3	0.003	0.29	0.8307
		Error	36	0.010		
10^{-4} Dilution	Females	Line	3	0.010	0.30	0.8228
		Error	36	0.030		
	Males	Line	3	0.005	0.22	0.8835
		Error	36	0.020		
**4-vinyl guaiacol**						
10^{-2} Dilution	Females	Line	3	0.020	1.64	0.1962
		Error	36	0.010		
	Males	Line	3	0.009	1.65	0.1942
		Error	36	0.005		
10^{-3} Dilution	Females	Line	3	0.030	1.76	0.1730
		Error	36	0.010		
	Males	Line	3	0.000	0.06	0.9806
		Error	36	0.006		
10^{-4} Dilution	Females	Line	3	0.010	0.75	0.5314
		Error	36	0.010		
	Males	Line	3	0.004	1.07	0.3728
		Error	36	0.004		

**Phenethyl acetate**						
10^{-2} Dilution	Females	Line	3	0.087	3.23	0.0340
		Error	35	0.027		
	Males	Line	3	0.009	0.75	0.5270
		Error	35	0.012		
10^{-3} Dilution	Females	Line	3	0.357	10.19	0.0001
		Error	28	0.035		
	Males	Line	3	0.035	3.16	0.0400
		Error	28	0.011		
10^{-4} Dilution	Females	Line	3	0.063	2.46	0.0790
		Error	34	0.025		
	Males	Line	3	0.023	1.12	0.3550
		Error	35	0.020		
