# Supplementary material for: Divergence in Olfactory Host Plant Preference in D. mojavensis in Response to Cactus Host Use
Source: PLoS One. 2013 Jul 25;8(7):e70027. doi: 10.1371/journal.pone.0070027 (PMC3723661; doi:10.1371/journal.pone.0070027)
Supplement: Table S2 — Relative amounts of volatile compounds in uninoculated and inoculated cacti. Volatile compounds (mean ± stdev) emitted from barrel, prickly pear, organ pipe and agria cacti. (PDF) [file pone.0070027.s003.pdf]

Table S2. Relative amounts of volatile compounds in uninoculated and inoculated cacti. Volatile compounds (mean ± stdev) emitted from barrel, prickly pear, organ pipe and agria cacti, respectively.

	Compound	Class	Uninoculated Barrel cactus	Week one Barrel cactus rot	Week two Barrel cactus rot	Week three Barrel cactus rot	Week four Barrel cactus rot
1	Acetone	ketone	0 ± 0	0 ± 0	4.83 ± 1.29	9.48 ± 13.40	8.70 ± 12.25
2	2-butanone	ketone	0 ± 0	0 ± 0	0 ± 0	0 ± 0	0 ± 0
3	2-propanol	alcohol	0 ± 0	0 ± 0	0 ± 0	0 ± 0	0 ± 0
4	Propyl acetate	ester	0 ± 0	0 ± 0	14.71 ± 0.37	12.21 ± 17.26	0 ± 0
5	Isopropyl acetate	ester	0 ± 0	0 ± 0	0 ± 0	10.29 ± 14.56	0 ± 0
6	Ethyl propionate	ester	0 ± 0	0 ± 0	0 ± 0	0 ± 0	0 ± 0
7	Isopropyl propionate	ester	0 ± 0	0 ± 0	0 ± 0	4.75 ± 6.71	10.44 ± 14.76
8	Propyl propionate	ester	0 ± 0	0 ± 0	7.02 ± 1.03	2.37 ± 3.35	0 ± 0
9	6-methyl-2 Hepatanone	ketone	0 ± 0	0 ± 0	0 ± 0	0 ± 0	0 ± 0
10	Hexanal	other	0 ± 0	0 ± 0	0 ± 0	0 ± 0	0 ± 0
11	Ethyl butyrate	ester	0 ± 0	0 ± 0	0 ± 0	0 ± 0	0 ± 0
12	Isopropyl isopentanoate	ester	0 ± 0	0 ± 0	0 ± 0	0 ± 0	0 ± 0
13	Isopropyl pentanoate	ester	0 ± 0	0 ± 0	0 ± 0	0 ± 0	0 ± 0
14	1-propanol	alcohol	0 ± 0	0 ± 0	0 ± 0	0 ± 0	0 ± 0
15	Isobutanol	alcohol	0 ± 0	0 ± 0	0 ± 0	0 ± 0	0 ± 0
16	Propyl butyrate	ester	0 ± 0	0 ± 0	0 ± 0	0 ± 0	0 ± 0
17	Isopentyl acetate	ester	0 ± 0	0 ± 0	11.30 ± 7.47	0.37 ± 0.52	13.77 ± 19.30
18	1-undecene	other	0 ± 0	1.85 ± 1.28	0 ± 0	0.09 ± 0.13	0 ± 0
19	1-dodecene	other	1.36 ± 1.86	0 ± 0	0 ± 0	0 ± 0	0 ± 0
20	Butyl propionate	ester	0 ± 0	0 ± 0	0 ± 0	0 ± 0	0 ± 0
21	Pentanoic acid 1-methylpropyl ester	ester	0 ± 0	0 ± 0	0 ± 0	0 ± 0	0 ± 0
22	Isopentyl propionate	ester	0 ± 0	0 ± 0	1.78 ± 2.51	1.82 ± 2.57	21.62 ± 18.77
23	2-heptanone	ketone	0.23 ± 0.02	0 ± 0	0 ± 0	0.39 ± 0.55	0 ± 0
24	Butyl butyrate	ester	0 ± 0	0 ± 0	0 ± 0	0 ± 0	0 ± 0
25	Isopentyl alcohol	alcohol	0.14 ± 0.19	0 ± 0	0.90 ± 0.18	0.96 ± 0.05	0 ± 0
26	Hexanoic acid ethyl ester	ester	0 ± 0	0 ± 0	0 ± 0	0 ± 0	0 ± 0
27	Hexanoic acid 1-methylethyl ester	ester	0 ± 0	0 ± 0	0 ± 0	0 ± 0	0 ± 0
28	Isopropyl tiglate	ester	0 ± 0	0 ± 0	0 ± 0	0 ± 0	0 ± 0

Table S2. Relative amounts of volatile compounds in uninoculated and inoculated cacti. Volatile compounds (mean ± stdev) emitted from barrel, prickly pear, organ pipe and agria cacti, respectively.

	Compound	Class	Uninoculated Barrel cactus	Week one Barrel cactus rot	Week two Barrel cactus rot	Week three Barrel cactus rot	Week four Barrel cactus rot
29	Isopentyl butyrate	ester	0 ± 0	0 ± 0	0 ± 0	0 ± 0	0 ± 0
30	2-heptanol acetate	ester	0 ± 0	0.12 ± 0.16	2.92 ± 1.39	1.40 ± 1.99	0 ± 0
31	3-octanone	ketone	0.35 ± 0.49	0 ± 0	0 ± 0	0 ± 0	0 ± 0
32	Hexyl acetate	ester	0 ± 0	1.42 ± 0.08	1.54 ± 0.82	0.56 ± 0.79	0.33 ± 0.46
33	Acetoin	ketone	0.58 ± 0.03	0 ± 0	0 ± 0	0 ± 0	0 ± 0
34	n-propyl hexanoate	ester	0 ± 0	0.10 ± 0.14	0 ± 0	0 ± 0	0.60 ± 0.84
35	Isobutyl tiglate	ester	0 ± 0	0 ± 0	0 ± 0	0 ± 0	0 ± 0
36	Hexyl propionate	ester	0 ± 0	0 ± 0	0 ± 0	0 ± 0	0 ± 0
37	2-methyl, 3-nonanol	alcohol	0 ± 0	0 ± 0	0 ± 0	0 ± 0	0 ± 0
38	1-hexanol	alcohol	0 ± 0	0.43 ± 0.04	0 ± 0	0.17 ± 0.24	0 ± 0
39	2-nonanone	ketone	1.46 ± 1.93	1.50 ± 1.18	2.94 ± 3.02	2.55 ± 2.78	1.39 ± 1.96
40	Durenol	aromatic	0 ± 0	2.87 ± 0.40	0 ± 0	0 ± 0	0 ± 0
41	Butyl hexanoate	ester	0 ± 0	0 ± 0	0 ± 0	0 ± 0	0 ± 0
42	Ethyl octanoate	ester	0 ± 0	0 ± 0	0 ± 0	0 ± 0	0 ± 0
43	Isopentyl hexanoate	ester	0 ± 0	0 ± 0	0 ± 0	0 ± 0	0 ± 0
44	2-octanol acetate	ester	0 ± 0	0 ± 0	0 ± 0	0 ± 0	0 ± 0
45	N,N'-diethyl-1,3 benzenediamine	aromatic	0 ± 0	0.77 ± 0.39	0 ± 0	1.01 ± 1.43	1.21 ± 0.85
46	2-nonanol	alcohol	0 ± 0	2.91 ± 1.06	1.73 ± 0.03	1.81 ± 1.72	0 ± 0
47	Propyl octanoate	ester	0 ± 0	0 ± 0	0 ± 0	0 ± 0	0 ± 0
48	Linalool	other	0 ± 0	0 ± 0	0.08 ± 0.12	0.03 ± 0.04	0 ± 0
49	Benzaldehyde	aromatic	0.35 ± 0.50	0 ± 0	0 ± 0	0 ± 0	0 ± 0
50	Acetic acid	acid	0 ± 0	0 ± 0	0 ± 0	0 ± 0	0 ± 0
51	Propionic acid	acid	0 ± 0	0 ± 0	0 ± 0	0 ± 0	0 ± 0
52	n-propyl, 3-mercapto-propanoate	ester	0 ± 0	0 ± 0	0 ± 0	0 ± 0	0 ± 0
53	Methyl benzoate	aromatic	0 ± 0	0.63 ± 0.30	2.60 ± 0.08	0.41 ± 0.37	1.10 ± 1.56
54	Butyric acid	acid	0 ± 0	0 ± 0	0 ± 0	0 ± 0	0 ± 0
55	Eugenol	aromatic	0 ± 0	0 ± 0	0 ± 0	0 ± 0	0 ± 0
56	Isopropyl benzoate	aromatic	0 ± 0	0 ± 0	1.34 ± 0.49	0.98 ± 1.38	1.87 ± 1.83
57	Acetophenone	aromatic	0 ± 0	0 ± 0	0 ± 0	0 ± 0	0 ± 0
58	Ethyl benzoate	aromatic	0 ± 0	4.07 ± 2.18	4.59 ± 0.93	7.37 ± 6.19	0.79 ± 1.11
59	Propyl benzoate	aromatic	0 ± 0	6.41 ± 2.28	2.23 ± 0.94	1.21 ± 0.26	0.37 ± 0.53
60	Pentanoic acid	acid	0 ± 0	0 ± 0	0 ± 0	0 ± 0	0 ± 0
61	Butyl benzoate	aromatic	0 ± 0	0 ± 0	0 ± 0	0 ± 0	0 ± 0
62	Methyl salicylate	aromatic	0 ± 0	0.31 ± 0.23	0 ± 0	0 ± 0	0 ± 0
63	2-tridecanone	ketone	0 ± 0	0.22 ± 0.09	0.48 ± 0.04	0.42 ± 0.15	0.50 ± 0.19
64	2-phenethyl acetate	aromatic	0 ± 0	0.14 ± 0.01	2.44 ± 0.06	1.80 ± 2.14	4.91 ± 3.94
65	2-methoxy phenol	aromatic	0 ± 0	6.03 ± 0.41	5.96 ± 3.69	4.34 ± 1.00	3.38 ± 1.90
66	Hexanoic acid	acid	0 ± 0	0 ± 0	0 ± 0	0 ± 0	0 ± 0

Table S2. Relative amounts of volatile compounds in uninoculated and inoculated cacti. Volatile compounds (mean ± stdev) emitted from barrel, prickly pear, organ pipe and agria cacti, respectively.

	Compound	Class	Uninoculated Barrel cactus	Week one Barrel cactus rot	Week two Barrel cactus rot	Week three Barrel cactus rot	Week four Barrel cactus rot
67	Phenethyl propionate	aromatic	0 ± 0	0 ± 0	0.85 ± 0.07	0.68 ± 0.96	8.40 ± 2.19
68	Isopentyl benzoate	aromatic	0 ± 0	0 ± 0	0 ± 0	0 ± 0	0 ± 0
69	Phenethyl alcohol	aromatic	0 ± 0	0.11 ± 0.01	0.11 ± 0.16	0.23 ± 0.16	0.31 ± 0.26
70	Creosol	aromatic	0 ± 0	0.24 ± 0.11	0.21 ± 0.12	0.25 ± 0.19	0.16 ± 0.14
71	Phenol	aromatic	0 ± 0	2.45 ± 0.08	2.19 ± 1.10	2.00 ± 1.58	1.61 ± 0.79
72	4-ethylguaiacol	aromatic	0 ± 0	2.97 ± 0.91	1.80 ± 1.66	1.75 ± 1.22	3.91 ± 2.24
73	4-methyl phenol	aromatic	0 ± 0	0.10 ± 0.00	0.13 ± 0.05	0.11 ± 0.06	0.13 ± 0.06
74	Octanoic acid	acid	0 ± 0	0 ± 0	0 ± 0	0 ± 0	0 ± 0
75	4-ethyl phenol	aromatic	0 ± 0	0.22 ± 0.16	0.03 ± 0.04	0.06 ± 0.09	0.18 ± 0.07
76	2-methoxy-4-propyl phenol	aromatic	0 ± 0	0.04 ± 0.06	0.05 ± 0.07	0 ± 0	0.01 ± 0.02
77	4-vinylguaiacol	aromatic	0 ± 0	0.18 ± 0.02	0.10 ± 0.03	0 ± 0	0.08 ± 0.11

Table S2. Relative amounts of volatile compounds in uninoculated and inoculated cacti. Volatile compounds (mean ± stdev) emitted from barrel, prickly pear, organ pipe and agria cacti, respectively.

	Compound	Week five Barrel cactus rot	Week six Barrel cactus rot	Week seven Barrel cactus rot	Week eight Barrel cactus rot	Week nine Barrel cactus rot
1	Acetone	6.16 ± 8.71	4.83 ± 1.91	15.72 ± 1.50	9.97 ± 4.18	14.51 ± 20.52
2	2-butanone	4.25 ± 0.51	0 ± 0	1.88 ± 2.66	0 ± 0	5.07 ± 7.17
3	2-propanol	0 ± 0	0 ± 0	0 ± 0	0 ± 0	0 ± 0
4	Propyl acetate	0 ± 0	0 ± 0	0 ± 0	0 ± 0	0 ± 0
5	Isopropyl acetate	0 ± 0	5.82 ± 3.06	0 ± 0	8.68 ± 12.27	0 ± 0
6	Ethyl propionate	0 ± 0	0 ± 0	0 ± 0	0 ± 0	0 ± 0
7	Isopropyl propionate	0 ± 0	7.92 ± 1.43	40.11 ± 51.59	17.73 ± 10.52	0 ± 0
8	Propyl propionate	0 ± 0	0 ± 0	0 ± 0	0 ± 0	0.38 ± 0.54
9	6-methyl-2 Hepatanone	0 ± 0	0 ± 0	0 ± 0	0 ± 0	0 ± 0
10	Hexanal	0 ± 0	0 ± 0	0 ± 0	0 ± 0	0 ± 0
11	Ethyl butyrate	0 ± 0	0 ± 0	0 ± 0	0 ± 0	0 ± 0
12	Isopropyl isopentanoate	0 ± 0	0 ± 0	0 ± 0	0 ± 0	0 ± 0
13	Isopropyl pentanoate	0 ± 0	0 ± 0	0 ± 0	0 ± 0	0 ± 0
14	1-propanol	0 ± 0	0 ± 0	0 ± 0	0 ± 0	0 ± 0
15	Isobutanol	0.09 ± 0.12	0.76 ± 1.07	0 ± 0	0 ± 0	0 ± 0
16	Propyl butyrate	0 ± 0	0 ± 0	0 ± 0	0 ± 0	0 ± 0
17	Isopentyl acetate	2.04 ± 2.53	9.27 ± 0.78	12.40 ± 14.44	15.64 ± 8.96	0.34 ± 0.48
18	1-undecene	0 ± 0	0 ± 0	0 ± 0	0 ± 0	0 ± 0
19	1-dodecene	0 ± 0	0 ± 0	0 ± 0	0 ± 0	0 ± 0
20	Butyl propionate	0 ± 0	0 ± 0	0 ± 0	0 ± 0	0 ± 0
21	Pentanoic acid 1-methylpropyl ester	0 ± 0	0 ± 0	0 ± 0	0 ± 0	0 ± 0
22	Isopentyl propionate	2.60 ± 0.02	17.28 ± 11.61	41.99 ± 53.67	13.99 ± 17.87	0.32 ± 0.08
23	2-heptanone	0 ± 0	0 ± 0	0 ± 0	0 ± 0	0 ± 0
24	Butyl butyrate	0 ± 0	0 ± 0	0 ± 0	0 ± 0	0 ± 0
25	Isopentyl alcohol	13.43 ± 14.32	0.56 ± 0.80	1.56 ± 2.21	1.56 ± 0.05	0.91 ± 0.65
26	Hexanoic acid ethyl ester	0 ± 0	0 ± 0	0 ± 0	0 ± 0	0 ± 0
27	Hexanoic acid 1-methylethyl ester	0 ± 0	0 ± 0	0 ± 0	0 ± 0	0 ± 0
28	Isopropyl tiglate	0 ± 0	0 ± 0	0 ± 0	0 ± 0	0 ± 0

Table S2. Relative amounts of volatile compounds in uninoculated and inoculated cacti. Volatile compounds (mean ± stdev) emitted from barrel, prickly pear, organ pipe and agria cacti, respectively.

	Compound	Week five Barrel cactus rot	Week six Barrel cactus rot	Week seven Barrel cactus rot	Week eight Barrel cactus rot	Week nine Barrel cactus rot
29	Isopentyl butyrate	0.18 ± 0.25	0 ± 0	0 ± 0	0 ± 0	0 ± 0
30	2-heptanol acetate	0 ± 0	0 ± 0	0 ± 0	0 ± 0	0 ± 0
31	3-octanone	0.12 ± 0.17	0 ± 0	0 ± 0	0 ± 0	0 ± 0
32	Hexyl acetate	0 ± 0	0 ± 0	0.13 ± 0.19	0 ± 0	0.27 ± 0.38
33	Acetoin	0.83 ± 0.59	0 ± 0	0 ± 0	0 ± 0	1.77 ± 1.51
34	n-propyl hexanoate	0 ± 0	0 ± 0	0 ± 0	0 ± 0	0 ± 0
35	Isobutyl tiglate	0 ± 0	0 ± 0	0 ± 0	0 ± 0	0 ± 0
36	Hexyl propionate	0 ± 0	0 ± 0	0 ± 0	0 ± 0	0 ± 0
37	2-methyl, 3-nonanol	0 ± 0	0 ± 0	0 ± 0	0 ± 0	0 ± 0
38	1-hexanol	0 ± 0	0 ± 0	0 ± 0	0 ± 0	0 ± 0
39	2-nonanone	1.75 ± 1.31	0.54 ± 0.77	1.43 ± 2.03	1.43 ± 1.12	0.12 ± 0.17
40	Durenol	0.68 ± 0.96	0.37 ± 0.52	0.53 ± 0.74	0 ± 0	3.07 ± 2.40
41	Butyl hexanoate	0 ± 0	0 ± 0	0 ± 0	0 ± 0	0 ± 0
42	Ethyl octanoate	0 ± 0	0 ± 0	0 ± 0	0 ± 0	0 ± 0
43	Isopentyl hexanoate	0 ± 0	0 ± 0	0 ± 0	0 ± 0	0 ± 0
44	2-octanol acetate	0 ± 0	0 ± 0	0 ± 0	0 ± 0	0 ± 0
45	N,N'-diethyl-1,3 benzenediamine	1.40 ± 1.98	1.42 ± 1.54	1.73 ± 1.62	0.72 ± 1.01	2.71 ± 0.91
46	2-nonanol	0.41 ± 0.01	0 ± 0	0.20 ± 0.14	0.10 ± 0.02	0 ± 0
47	Propyl octanoate	0 ± 0	0 ± 0	0 ± 0	0 ± 0	0 ± 0
48	Linalool	0 ± 0	0 ± 0	0 ± 0	0 ± 0	0.06 ± 0.08
49	Benzaldehyde	0 ± 0	0 ± 0	0 ± 0	0 ± 0	0.05 ± 0.06
50	Acetic acid	0 ± 0	0 ± 0	0 ± 0	0 ± 0	0 ± 0
51	Propionic acid	0 ± 0	0 ± 0	0 ± 0	0 ± 0	0 ± 0
52	n-propyl, 3-mercapto-propanoate	0 ± 0	0 ± 0	0 ± 0	0 ± 0	0 ± 0
53	Methyl benzoate	5.02 ± 3.94	1.70 ± 1.61	1.03 ± 1.45	1.30 ± 0.56	0 ± 0
54	Butyric acid	0 ± 0	0 ± 0	0 ± 0	0 ± 0	0 ± 0
55	Eugenol	0.20 ± 0.28	0 ± 0	0 ± 0	0 ± 0	0 ± 0
56	Isopropyl benzoate	1.22 ± 1.73	1.36 ± 0.68	0.95 ± 0.62	0.78 ± 0.63	0 ± 0
57	Acetophenone	0.16 ± 0.23	0 ± 0	0 ± 0	0.11 ± 0.16	0.11 ± 0.16
58	Ethyl benzoate	0.32 ± 0.46	0 ± 0	0.18 ± 0.25	0.08 ± 0.11	0 ± 0
59	Propyl benzoate	0 ± 0	0 ± 0	0.25 ± 0.36	0.24 ± 0.34	0 ± 0
60	Pentanoic acid	0 ± 0	0 ± 0	0 ± 0	0 ± 0	0 ± 0
61	Butyl benzoate	0.37 ± 0.52	0.27 ± 0.38	0.40 ± 0.56	0 ± 0	0 ± 0
62	Methyl salicylate	0.10 ± 0.05	0 ± 0	0 ± 0	0 ± 0	0 ± 0
63	2-tridecanone	0.29 ± 0.04	0.22 ± 0.32	0 ± 0	0.33 ± 0.09	0.11 ± 0.15
64	2-phenethyl acetate	1.71 ± 2.05	3.37 ± 3.44	3.28 ± 3.50	3.44 ± 0.08	0.65 ± 0.14
65	2-methoxy phenol	5.96 ± 1.31	2.97 ± 1.09	4.20 ± 1.64	2.68 ± 1.10	12.14 ± 2.87
66	Hexanoic acid	0.14 ± 0.20	0 ± 0	0 ± 0	0 ± 0	0 ± 0

Table S2. Relative amounts of volatile compounds in uninoculated and inoculated cacti. Volatile compounds (mean ± stdev) emitted from barrel, prickly pear, organ pipe and agria cacti, respectively.

	**Compound**	**Week five Barrel cactus rot**	**Week six Barrel cactus rot**	**Week seven Barrel cactus rot**	**Week eight Barrel cactus rot**	**Week nine Barrel cactus rot**
67	Phenethyl propionate	0.79 ± 0.58	7.38 ± 7.07	9.97 ± 11.03	7.15 ± 2.21	0.23 ± 0.32
68	Isopentyl benzoate	1.86 ± 2.15	0 ± 0	0.12 ± 0.16	0.09 ± 0.13	0 ± 0
69	Phenethyl alcohol	3.79 ± 1.48	0.22 ± 0.31	0.18 ± 0.26	0.32 ± 0.08	1.19 ± 0.24
70	Creosol	0.46 ± 0.37	0.42 ± 0.39	0.82 ± 0.91	1.38 ± 1.69	0.25 ± 0.08
71	Phenol	1.64 ± 0.57	1.78 ± 0.16	2.45 ± 0.42	1.91 ± 0.08	2.29 ± 0.74
72	4-ethylguaiacol	63.07 ± 35.29	2.98 ± 2.09	10.05 ± 7.92	2.43 ± 1.14	115.55 ± 105.80
73	4-methyl phenol	0.09 ± 0.01	0.21 ± 0.11	0.16 ± 0.08	0.17 ± 0.04	0.23 ± 0.01
74	Octanoic acid	0 ± 0	0 ± 0	0 ± 0	0 ± 0	0 ± 0
75	4-ethyl phenol	0.15 ± 0.04	0.13 ± 0.19	0.03 ± 0.05	0 ± 0	0.65 ± 0.60
76	2-methoxy-4-propyl phenol	0.16 ± 0.23	0 ± 0	0.14 ± 0.04	0.11 ± 0.02	0.22 ± 0.31
77	4-vinylguaiacol	0.13 ± 0.02	0.26 ± 0.13	0.14 ± 0.04	0.07 ± 0.10	0.33 ± 0.30

Table S2. Relative amounts of volatile compounds in uninoculated and inoculated cacti. Volatile compounds (mean ± stdev) emitted from barrel, prickly pear, organ pipe and agria cacti, respectively.

	Compound	Uninoculated Prickly pear cactus	Week one Prickly pear cactus rot	Week two Prickly pear cactus rot	Week three Prickly pear cactus rot	Week four Prickly pear cactus rot
1	Acetone	0 ± 0	0.57 ± 0.81	2.44 ± 2.53	3.98 ± 1.36	7.72 ± 8.65
2	2-butanone	0 ± 0	0 ± 0	11.14 ± 3.54	18.83 ± 23.21	4.36 ± 0.45
3	2-propanol	0 ± 0	0 ± 0	0 ± 0	0 ± 0	0 ± 0
4	Propyl acetate	0 ± 0	0 ± 0	0 ± 0	0 ± 0	0 ± 0
5	Isopropyl acetate	0 ± 0	0 ± 0	0 ± 0	0 ± 0	0 ± 0
6	Ethyl propionate	0 ± 0	0 ± 0	3.38E-07 ± 0.00	0 ± 0	0 ± 0
7	Isopropyl propionate	0 ± 0	0 ± 0	0 ± 0	0 ± 0	0 ± 0
8	Propyl propionate	0 ± 0	0 ± 0	19.42 ± 27.46	0 ± 0	0 ± 0
9	6-methyl-2 Hepatanone	0 ± 0	0 ± 0	0 ± 0	0 ± 0	0 ± 0
10	Hexanal	0 ± 0	0 ± 0	0 ± 0	0 ± 0	0 ± 0
11	Ethyl butyrate	0 ± 0	6.04 ± 0.09	0 ± 0	0 ± 0	0 ± 0
12	Isopropyl isopentanoate	0 ± 0	0 ± 0	0 ± 0	0 ± 0	0 ± 0
13	Isopropyl pentanoate	0 ± 0	0 ± 0	0 ± 0	0 ± 0	0 ± 0
14	1-propanol	0 ± 0	0 ± 0	0 ± 0	0 ± 0	0 ± 0
15	Isobutanol	0.19 ± 0.27	0 ± 0	0 ± 0	0 ± 0	0 ± 0
16	Propyl butyrate	0 ± 0	0 ± 0	0 ± 0	0 ± 0	0 ± 0
17	Isopentyl acetate	0 ± 0	0 ± 0	9.30 ± 13.16	0.69 ± 0.97	0 ± 0
18	1-undecene	0 ± 0	0 ± 0	0 ± 0	0 ± 0	0 ± 0
19	1-dodecene	0 ± 0	0 ± 0	0 ± 0	0 ± 0	0 ± 0
20	Butyl propionate	0 ± 0	0 ± 0	0 ± 0	0 ± 0	0 ± 0
21	Pentanoic acid 1-methylpropyl ester	0 ± 0	0 ± 0	0 ± 0	0 ± 0	0 ± 0
22	Isopentyl propionate	0 ± 0	0 ± 0	14.43 ± 20.41	1.31 ± 1.85	0 ± 0
23	2-heptanone	1.07 ± 0.06	0 ± 0	0.11 ± 0.15	0 ± 0	0 ± 0
24	Butyl butyrate	5.82 ± 0.16	12.07 ± 2.26	0 ± 0	0 ± 0	0 ± 0
25	Isopentyl alcohol	0 ± 0	0.82 ± 1.17	1.36 ± 1.05	2.28 ± 3.23	0.17 ± 0.24
26	Hexanoic acid ethyl ester	0 ± 0	2.00 ± 0.66	0 ± 0	0 ± 0	0 ± 0
27	Hexanoic acid 1-methylethyl ester	0 ± 0	0.65 ± 0.93	0 ± 0	0 ± 0	1.50 ± 0.84
28	Isopropyl tiglate	0 ± 0	0 ± 0	0 ± 0	0 ± 0	0 ± 0

Table S2. Relative amounts of volatile compounds in uninoculated and inoculated cacti. Volatile compounds (mean ± stdev) emitted from barrel, prickly pear, organ pipe and agria cacti, respectively.

	Compound	Uninoculated Prickly pear cactus	Week one Prickly pear cactus rot	Week two Prickly pear cactus rot	Week three Prickly pear cactus rot	Week four Prickly pear cactus rot
29	Isopentyl butyrate	0 ± 0	7.16 ± 0.27	0 ± 0	0 ± 0	0 ± 0
30	2-heptanol acetate	0 ± 0	0 ± 0	0 ± 0	0 ± 0	0 ± 0
31	3-octanone	1.23 ± 0.57	0 ± 0	0 ± 0	0 ± 0	0 ± 0
32	Hexyl acetate	0 ± 0	0.65 ± 0.92	0.54 ± 0.76	0 ± 0	0 ± 0
33	Acetoin	3.29 ± 1.42	0.33 ± 0.47	0.62 ± 0.88	0.10 ± 0.15	0 ± 0
34	n-propyl hexanoate	0 ± 0	0.68 ± 0.41	0 ± 0	0 ± 0	0 ± 0
35	Isobutyl tiglate	0 ± 0	0 ± 0	0 ± 0	0 ± 0	0 ± 0
36	Hexyl propionate	0 ± 0	0 ± 0	0 ± 0	0 ± 0	0 ± 0
37	2-methyl, 3-nonanol	0 ± 0	0 ± 0	0 ± 0	0 ± 0	0 ± 0
38	1-hexanol	1.01 ± 0.37	0.14 ± 0.20	0 ± 0	0 ± 0	0 ± 0
39	2-nonanone	9.13 ± 0.86	0.23 ± 0.16	0 ± 0	0.37 ± 0.52	0 ± 0
40	Durenol	0 ± 0	0 ± 0	0 ± 0	0 ± 0	0 ± 0
41	Butyl hexanoate	0 ± 0	1.74 ± 1.77	0 ± 0	0 ± 0	0 ± 0
42	Ethyl octanoate	0 ± 0	0 ± 0	0 ± 0	0 ± 0	0 ± 0
43	Isopentyl hexanoate	0 ± 0	0.69 ± 0.62	0 ± 0	0 ± 0	0 ± 0
44	2-octanol acetate	0 ± 0	0 ± 0	0 ± 0	0 ± 0	0 ± 0
45	N,N'-diethyl-1,3 benzenediamine	0 ± 0	0 ± 0	0 ± 0	0 ± 0	0 ± 0
46	2-nonanol	1.21 ± 0.11	0 ± 0	0 ± 0	0.07 ± 0.09	0 ± 0
47	Propyl octanoate	0 ± 0	0 ± 0	0 ± 0	0 ± 0	0 ± 0
48	Linalool	1.22 ± 0.50	5.72 ± 0.24	0.98 ± 0.55	1.49 ± 0.35	8.34 ± 6.09
49	Benzaldehyde	0 ± 0	0 ± 0	0 ± 0	0 ± 0	0 ± 0
50	Acetic acid	0 ± 0	0 ± 0	0 ± 0	0 ± 0	0 ± 0
51	Propionic acid	0 ± 0	0 ± 0	0 ± 0	0 ± 0	0 ± 0
52	n-propyl, 3-mercapto-propanoate	0 ± 0	0 ± 0	0 ± 0	0 ± 0	0 ± 0
53	Methyl benzoate	0 ± 0	0.17 ± 0.24	0.17 ± 0.24	0.21 ± 0.30	0 ± 0
54	Butyric acid	0 ± 0	50.13 ± 20.58	0 ± 0	0 ± 0	0 ± 0
55	Eugenol	0 ± 0	0 ± 0	0 ± 0	0 ± 0	0 ± 0
56	Isopropyl benzoate	0 ± 0	5.46 ± 3.06	0.08 ± 0.11	0.45 ± 0.38	0.70 ± 0.23
57	Acetophenone	0 ± 0	0 ± 0	0 ± 0	0 ± 0	0.04 ± 0.05
58	Ethyl benzoate	0 ± 0	2.62 ± 1.70	1.30 ± 1.84	0.20 ± 0.09	0 ± 0
59	Propyl benzoate	0 ± 0	0.14 ± 0.04	0.68 ± 0.96	0.14 ± 0.02	0 ± 0
60	Pentanoic acid	0 ± 0	0 ± 0	0 ± 0	0 ± 0	0 ± 0
61	Butyl benzoate	0 ± 0	0.03 ± 0.04	0 ± 0	0 ± 0	0 ± 0
62	Methyl salicylate	0.13 ± 0.03	3.07 ± 0.41	0.37 ± 0.53	0.27 ± 0.16	0.21 ± 0.29
63	2-tridecanone	0.65 ± 0.16	0 ± 0	0 ± 0	0 ± 0	0 ± 0
64	2-phenethyl acetate	0 ± 0	1.03 ± 1.24	0 ± 0	2.75 ± 0.79	0.38 ± 0.54
65	2-methoxy phenol	0.13 ± 0.11	0 ± 0	0.18 ± 0.25	0 ± 0	0 ± 0
66	Hexanoic acid	0 ± 0	7.82 ± 4.93	0 ± 0	0 ± 0	0 ± 0

Table S2. Relative amounts of volatile compounds in uninoculated and inoculated cacti. Volatile compounds (mean ± stdev) emitted from barrel, prickly pear, organ pipe and agria cacti, respectively.

	Compound	Uninoculated Prickly pear cactus	Week one Prickly pear cactus rot	Week two Prickly pear cactus rot	Week three Prickly pear cactus rot	Week four Prickly pear cactus rot
67	Phenethyl propionate	0 ± 0	0.51 ± 0.71	9.11 ± 12.88	2.26 ± 0.13	0 ± 0
68	Isopentyl benzoate	0 ± 0	0.10 ± 0.14	0.08 ± 0.12	0.43 ± 0.60	0 ± 0
69	Phenethyl alcohol	0.31 ± 0.05	0.59 ± 0.36	0.21 ± 0.08	5.82 ± 1.22	0.37 ± 0.35
70	Creosol	0 ± 0	0 ± 0	0.02 ± 0.03	0.03 ± 0.04	0 ± 0
71	Phenol	0.42 ± 0.59	0.68 ± 0.21	0.42 ± 0.26	0.57 ± 0.17	0.34 ± 0.23
72	4-ethylguaiacol	0.03 ± 0.04	0.08 ± 0.00	0.69 ± 0.98	0 ± 0	0 ± 0
73	4-methyl phenol	0 ± 0	13.88 ± 19.19	0.93 ± 1.31	3.48 ± 4.80	23.73 ± 28.71
74	Octanoic acid	0 ± 0	0 ± 0	0 ± 0	0 ± 0	0 ± 0
75	4-ethyl phenol	0 ± 0	1.06 ± 0.25	0 ± 0	0.44 ± 0.21	0.90 ± 1.06
76	2-methoxy-4-propyl phenol	0 ± 0	0 ± 0	0 ± 0	0 ± 0	0 ± 0
77	4-vinylguaiacol	0 ± 0	0 ± 0	0 ± 0	0 ± 0	0 ± 0

Table S2. Relative amounts of volatile compounds in uninoculated and inoculated cacti. Volatile compounds (mean ± stdev) emitted from barrel, prickly pear, organ pipe and agria cacti, respectively.

	**Compound**	**Week five Prickly pear cactus rot**	**Week six Prickly pear cactus rot**	**Week seven Prickly pear cactus rot**	**Week eight Prickly pear cactus rot**	**Week nine Prickly pear cactus rot**
1	Acetone	12.47 ± 15.84	12.50 ± 4.96	9.45 ± 1.33	2.26 ± 3.19	19.43 ± 27.48
2	2-butanone	11.48 ± 12.33	4.74 ± 6.70	14.77 ± 18.43	0 ± 0	4.44 ± 6.28
3	2-propanol	0 ± 0	0 ± 0	0 ± 0	0 ± 0	0 ± 0
4	Propyl acetate	0 ± 0	0 ± 0	0 ± 0	0 ± 0	0 ± 0
5	Isopropyl acetate	0 ± 0	0 ± 0	0 ± 0	0 ± 0	0 ± 0
6	Ethyl propionate	0 ± 0	0 ± 0	0 ± 0	0 ± 0	0 ± 0
7	Isopropyl propionate	0 ± 0	0 ± 0	0 ± 0	0 ± 0	0 ± 0
8	Propyl propionate	0 ± 0	0 ± 0	0 ± 0	0 ± 0	0 ± 0
9	6-methyl-2 Hepatanone	0 ± 0	0 ± 0	0 ± 0	0 ± 0	0 ± 0
10	Hexanal	0 ± 0	0 ± 0	0 ± 0	0 ± 0	0 ± 0
11	Ethyl butyrate	0 ± 0	0 ± 0	0 ± 0	0 ± 0	0 ± 0
12	Isopropyl isopentanoate	0.76 ± 1.07	0 ± 0	0 ± 0	0 ± 0	0 ± 0
13	Isopropyl pentanoate	2.58 ± 3.65	0 ± 0	0 ± 0	0 ± 0	0 ± 0
14	1-propanol	0 ± 0	0 ± 0	0 ± 0	0 ± 0	0 ± 0
15	Isobutanol	0 ± 0	0 ± 0	0 ± 0	0 ± 0	37.58 ± 53.15
16	Propyl butyrate	0 ± 0	0 ± 0	0 ± 0	0 ± 0	0 ± 0
17	Isopentyl acetate	0.42 ± 0.59	0 ± 0	0 ± 0	0 ± 0	0 ± 0
18	1-undecene	0 ± 0	0 ± 0	0 ± 0	0 ± 0	0 ± 0
19	1-dodecene	0 ± 0	0 ± 0	0 ± 0	0 ± 0	0 ± 0
20	Butyl propionate	0 ± 0	0 ± 0	0 ± 0	0 ± 0	0 ± 0
21	Pentanoic acid 1-methylpropyl ester	0.29 ± 0.41	0 ± 0	0 ± 0	0 ± 0	0 ± 0
22	Isopentyl propionate	0.04 ± 0.05	0 ± 0	0 ± 0	0 ± 0	3.46 ± 4.44
23	2-heptanone	0 ± 0	0 ± 0	0 ± 0	0 ± 0	0 ± 0
24	Butyl butyrate	0 ± 0	0 ± 0	0 ± 0	0 ± 0	0 ± 0
25	Isopentyl alcohol	0.78 ± 1.11	0.14 ± 0.19	0.36 ± 0.20	0 ± 0	10.44 ± 0.19
26	Hexanoic acid ethyl ester	0 ± 0	0 ± 0	0 ± 0	0 ± 0	0 ± 0
27	Hexanoic acid 1-methylethyl ester	1.56 ± 2.20	0 ± 0	0 ± 0	0 ± 0	0 ± 0
28	Isopropyl tiglate	0.40 ± 0.13	0 ± 0	0 ± 0	0 ± 0	0 ± 0

Table S2. Relative amounts of volatile compounds in uninoculated and inoculated cacti. Volatile compounds (mean ± stdev) emitted from barrel, prickly pear, organ pipe and agria cacti, respectively.

	Compound	Week five Prickly pear cactus rot	Week six Prickly pear cactus rot	Week seven Prickly pear cactus rot	Week eight Prickly pear cactus rot	Week nine Prickly pear cactus rot
29	Isopentyl butyrate	0 ± 0	0 ± 0	0 ± 0	0 ± 0	0 ± 0
30	2-heptanol acetate	0 ± 0	0 ± 0	0 ± 0	0 ± 0	0.59 ± 0.84
31	3-octanone	0.10 ± 0.15	0 ± 0	0 ± 0	0 ± 0	0 ± 0
32	Hexyl acetate	0 ± 0	0 ± 0	0 ± 0	0 ± 0	0.14 ± 0.20
33	Acetoin	0 ± 0	0 ± 0	0 ± 0	0 ± 0	0.27 ± 0.38
34	n-propyl hexanoate	0 ± 0	0 ± 0	0 ± 0	0 ± 0	0 ± 0
35	Isobutyl tiglate	0.52 ± 0.74	0 ± 0	0 ± 0	0 ± 0	0 ± 0
36	Hexyl propionate	0 ± 0	0 ± 0	0 ± 0	0 ± 0	0 ± 0
37	2-methyl, 3-nonanol	0.12 ± 0.16	0 ± 0	0 ± 0	0 ± 0	0 ± 0
38	1-hexanol	0 ± 0	0 ± 0	0 ± 0	0 ± 0	0 ± 0
39	2-nonanone	0.14 ± 0.05	0.07 ± 0.09	0.07 ± 0.10	0 ± 0	0.73 ± 0.65
40	Durenol	0 ± 0	0 ± 0	0 ± 0	0 ± 0	0 ± 0
41	Butyl hexanoate	0 ± 0	0 ± 0	0 ± 0	0 ± 0	0 ± 0
42	Ethyl octanoate	0 ± 0	0 ± 0	0 ± 0	0 ± 0	0 ± 0
43	Isopentyl hexanoate	0 ± 0	0 ± 0	0 ± 0	0 ± 0	0 ± 0
44	2-octanol acetate	0 ± 0	0 ± 0	0 ± 0	0 ± 0	0.09 ± 0.12
45	N,N'-diethyl-1,3 benzenediamine	0 ± 0	0 ± 0	0 ± 0	0 ± 0	0 ± 0
46	2-nonanol	0 ± 0	0 ± 0	0 ± 0	0 ± 0	0.20 ± 0.28
47	Propyl octanoate	0 ± 0	0 ± 0	0 ± 0	0 ± 0	0 ± 0
48	Linalool	0.55 ± 0.15	7.33 ± 0.93	3.81 ± 4.99	9.01 ± 6.54	0.27 ± 0.02
49	Benzaldehyde	0 ± 0	0 ± 0	0 ± 0	0 ± 0	0 ± 0
50	Acetic acid	0 ± 0	0 ± 0	0 ± 0	0 ± 0	0 ± 0
51	Propionic acid	0 ± 0	0 ± 0	0 ± 0	0 ± 0	0 ± 0
52	n-propyl, 3-mercapto-propanoate	0 ± 0	0 ± 0	0 ± 0	0 ± 0	0.66 ± 0.93
53	Methyl benzoate	0.16 ± 0.07	0 ± 0	0.51 ± 0.73	0 ± 0	0.68 ± 0.96
54	Butyric acid	0 ± 0	0 ± 0	0 ± 0	0 ± 0	0 ± 0
55	Eugenol	0.24 ± 0.34	0 ± 0	0 ± 0	0 ± 0	0 ± 0
56	Isopropyl benzoate	0.39 ± 0.55	0.08 ± 0.12	1.20 ± 0.36	0.12 ± 0.17	0.65 ± 0.91
57	Acetophenone	0.04 ± 0.06	0.03 ± 0.04	0.05 ± 0.07	0 ± 0	0 ± 0
58	Ethyl benzoate	0.70 ± 0.81	0 ± 0	0 ± 0	0.13 ± 0.18	0.25 ± 0.04
59	Propyl benzoate	0 ± 0	0 ± 0	0 ± 0	0.04 ± 0.06	0 ± 0
60	Pentanoic acid	0 ± 0	0 ± 0	0 ± 0	0 ± 0	0 ± 0
61	Butyl benzoate	0 ± 0	0 ± 0	0 ± 0	0 ± 0	0.10 ± 0.15
62	Methyl salicylate	1.07 ± 0.66	0.06 ± 0.08	0.04 ± 0.05	0 ± 0	2.66 ± 2.32
63	2-tridecanone	0 ± 0	0 ± 0	0.07 ± 0.09	0 ± 0	0.06 ± 0.09
64	2-phenethyl acetate	1.38 ± 1.37	0.07 ± 0.10	0.19 ± 0.27	0 ± 0	18.68 ± 26.11
65	2-methoxy phenol	0.22 ± 0.31	0 ± 0	0.36 ± 0.51	0 ± 0	0.13 ± 0.03
66	Hexanoic acid	0 ± 0	0 ± 0	0 ± 0	0 ± 0	0.12 ± 0.18

Table S2. Relative amounts of volatile compounds in uninoculated and inoculated cacti. Volatile compounds (mean ± stdev) emitted from barrel, prickly pear, organ pipe and agria cacti, respectively.

	Compound	Week five Prickly pear cactus rot	Week six Prickly pear cactus rot	Week seven Prickly pear cactus rot	Week eight Prickly pear cactus rot	Week nine Prickly pear cactus rot
67	Phenethyl propionate	0.32 ± 0.25	0 ± 0	0 ± 0	0 ± 0	1.71 ± 2.42
68	Isopentyl benzoate	0 ± 0	0 ± 0	0 ± 0	0 ± 0	0.26 ± 0.36
69	Phenethyl alcohol	2.89 ± 0.43	1.48 ± 1.68	1.78 ± 0.83	1.35 ± 0.86	1.60 ± 1.80
70	Creosol	0.10 ± 0.14	0 ± 0	0.01 ± 0.02	0 ± 0	0 ± 0
71	Phenol	0.76 ± 0.63	0.30 ± 0.12	0.34 ± 0.02	0.20 ± 0.07	0.59 ± 0.45
72	4-ethylguaiacol	0.46 ± 0.65	0.01 ± 0.01	0.18 ± 0.26	0 ± 0	1.49 ± 1.81
73	4-methyl phenol	1.88 ± 0.99	195.72 ± 65.71	86.73 ± 111.44	105.49 ± 34.48	0.07 ± 0.03
74	Octanoic acid	0 ± 0	0 ± 0	0 ± 0	0 ± 0	0 ± 0
75	4-ethyl phenol	0.38 ± 0.13	1.09 ± 0.22	2.06 ± 0.51	1.92 ± 0.52	0.75 ± 0.15
76	2-methoxy-4-propyl phenol	0 ± 0	0 ± 0	0 ± 0	0 ± 0	0 ± 0
77	4-vinylguaiacol	0 ± 0	0 ± 0	0 ± 0	0 ± 0	0.11 ± 0.16

Table S2. Relative amounts of volatile compounds in uninoculated and inoculated cacti. Volatile compounds (mean ± stdev) emitted from barrel, prickly pear, organ pipe and agria cacti, respectively.

	Compound	Uninoculated Organ Pipe cactus	Week one Organ Pipe cactus rot	Week two Organ Pipe cactus rot	Week three Organ Pipe cactus rot	Week four Organ Pipe cactus rot
1	Acetone	0 ± 0	0 ± 0	0 ± 0	2.33 ± 4.04	1.55 ± 1.35
2	2-butanone	0 ± 0	0 ± 0	0 ± 0	0 ± 0	0 ± 0
3	2-propanol	0 ± 0	3.84 ± 1.84	2.5 ± 1.12	0 ± 0	0 ± 0
4	Propyl acetate	0 ± 0	0 ± 0	7.21 ± 2.78	7.05 ± 3.3	7.57 ± 2.11
5	Isopropyl acetate	0 ± 0	0 ± 0	0 ± 0	0 ± 0	0 ± 0
6	Ethyl propionate	0 ± 0	0 ± 0	0 ± 0	0 ± 0	0 ± 0
7	Isopropyl propionate	0 ± 0	0 ± 0	0 ± 0	0 ± 0	0 ± 0
8	Propyl propionate	0 ± 0	0 ± 0	34.76 ± 8.11	48.67 ± 7.44	56.89 ± 8.61
9	6-methyl-2 Hepatanone	0 ± 0	0 ± 0	0 ± 0	0 ± 0	0 ± 0
10	Hexanal	0.33 ± 0.1	0 ± 0	0 ± 0	0 ± 0	0 ± 0
11	Ethyl butyrate	0 ± 0	2.02 ± 3.51	0.16 ± 0.27	0.12 ± 0.22	0 ± 0
12	Isopropyl isopentanoate	0 ± 0	0 ± 0	0 ± 0	0 ± 0	0 ± 0
13	Isopropyl pentanoate	0 ± 0	0 ± 0	0 ± 0	0 ± 0	0 ± 0
14	1-propanol	0 ± 0	2.27 ± 3.93	0 ± 0	0 ± 0	0 ± 0
15	Isobutanol	0 ± 0	0 ± 0	0 ± 0	0 ± 0	0 ± 0
16	Propyl butyrate	0 ± 0	3.1 ± 0.45	4.76 ± 1.85	4.21 ± 2.41	5.34 ± 1.75
17	Isopentyl acetate	0 ± 0	0 ± 0	0 ± 0	0 ± 0	0 ± 0
18	1-undecene	0 ± 0	0 ± 0	0 ± 0	0 ± 0	0.01 ± 0.01
19	1-dodecene	0 ± 0	0 ± 0	0 ± 0	0 ± 0	0 ± 0
20	Butyl propionate	0 ± 0	0 ± 0	0.89 ± 0.36	1.17 ± 0.56	1.65 ± 0.27
21	Pentanoic acid 1-methylpropyl ester	0 ± 0	0 ± 0	0 ± 0	0 ± 0	0 ± 0
22	Isopentyl propionate	0 ± 0	0 ± 0	0.3 ± 0.53	0.15 ± 0.27	0.86 ± 0.51
23	2-heptanone	0 ± 0	0 ± 0	0 ± 0	0 ± 0	0 ± 0
24	Butyl butyrate	0 ± 0	0 ± 0	0 ± 0	0 ± 0	0.22 ± 0.22
25	Isopentyl alcohol	0 ± 0	0 ± 0	0 ± 0	0 ± 0	0.07 ± 0.08
26	Hexanoic acid ethyl ester	0 ± 0	0.79 ± 0.37	0.53 ± 0.49	0.13 ± 0.23	0.63 ± 0.38
27	Hexanoic acid 1-methylethyl ester	0 ± 0	0.1 ± 0.17	0.17 ± 0.3	0 ± 0	0.21 ± 0.37
28	Isopropyl tiglate	0 ± 0	0 ± 0	0 ± 0	0 ± 0	0 ± 0

Table S2. Relative amounts of volatile compounds in uninoculated and inoculated cacti. Volatile compounds (mean ± stdev) emitted from barrel, prickly pear, organ pipe and agria cacti, respectively.

	Compound	Uninoculated Organ Pipe cactus	Week one Organ Pipe cactus rot	Week two Organ Pipe cactus rot	Week three Organ Pipe cactus rot	Week four Organ Pipe cactus rot
29	Isopentyl butyrate	0 ± 0	0 ± 0	0 ± 0	0 ± 0	0 ± 0
30	2-heptanol acetate	0 ± 0	0 ± 0	0 ± 0	0 ± 0	0 ± 0
31	3-octanone	0 ± 0	0 ± 0	0 ± 0	0 ± 0	0 ± 0
32	Hexyl acetate	0 ± 0	0.27 ± 0.07	0.62 ± 0.16	0.52 ± 0.28	0.64 ± 0.61
33	Acetoin	0 ± 0	0 ± 0	0 ± 0	0 ± 0	0 ± 0
34	n-propyl hexanoate	0 ± 0	1.35 ± 0.65	2.84 ± 0.71	3.09 ± 0.29	3.09 ± 0.69
35	Isobutyl tiglate	0 ± 0	0 ± 0	0 ± 0	0 ± 0	0 ± 0
36	Hexyl propionate	0 ± 0	0 ± 0	0.53 ± 0.55	0.61 ± 0.54	0.71 ± 0.76
37	2-methyl, 3-nonanol	0 ± 0	0 ± 0	0 ± 0	0 ± 0	0 ± 0
38	1-hexanol	0 ± 0	0.17 ± 0.02	0 ± 0	0 ± 0	0 ± 0
39	2-nonanone	0 ± 0	0 ± 0	0 ± 0	0 ± 0	0 ± 0
40	Durenol	0 ± 0	0 ± 0	0 ± 0	0 ± 0	0 ± 0
41	Butyl hexanoate	0 ± 0	0 ± 0	0.02 ± 0.04	0.03 ± 0.05	0 ± 0
42	Ethyl octanoate	0 ± 0	0.27 ± 0.24	0.44 ± 0.22	0.55 ± 0.06	0.26 ± 0.15
43	Isopentyl hexanoate	0 ± 0	0 ± 0	0.02 ± 0.04	0 ± 0	0 ± 0
44	2-octanol acetate	0 ± 0	0 ± 0	0 ± 0	0 ± 0	0 ± 0
45	N,N'-diethyl-1,3 benzenediamine	0 ± 0	0 ± 0	0 ± 0	0 ± 0	0 ± 0
46	2-nonanol	0 ± 0	0 ± 0	0 ± 0	0 ± 0	0 ± 0
47	Propyl octanoate	0 ± 0	0.36 ± 0.23	1.57 ± 1.32	1.31 ± 0.23	1.09 ± 0.43
48	Linalool	0 ± 0	0 ± 0	0 ± 0	0 ± 0	0 ± 0
49	Benzaldehyde	0.1 ± 0.06	0 ± 0	0 ± 0	0 ± 0	0 ± 0
50	Acetic acid	0 ± 0	0 ± 0	0 ± 0	0 ± 0	0 ± 0
51	Propionic acid	0 ± 0	0.77 ± 0.73	2.84 ± 1.09	2.84 ± 1.55	4.14 ± 1.33
52	n-propyl, 3-mercapto-propanoate	0 ± 0	0 ± 0	0 ± 0	0 ± 0	0 ± 0
53	Methyl benzoate	0 ± 0	0 ± 0	0 ± 0	0 ± 0	0 ± 0
54	Butyric acid	0 ± 0	26.61 ± 5.17	33.87 ± 15.51	26.44 ± 15.73	26.37 ± 9.04
55	Eugenol	0 ± 0	0 ± 0	0 ± 0	0 ± 0	0 ± 0
56	Isopropyl benzoate	0 ± 0	0 ± 0	0 ± 0	0 ± 0	0 ± 0
57	Acetophenone	0 ± 0	0 ± 0	0 ± 0	0 ± 0	0 ± 0
58	Ethyl benzoate	0 ± 0	0 ± 0	0 ± 0	0 ± 0	0 ± 0
59	Propyl benzoate	0 ± 0	0 ± 0	0 ± 0	0 ± 0	0.02 ± 0.04
60	Pentanoic acid	0 ± 0	0 ± 0	0 ± 0	0.87 ± 1.5	1.7 ± 2.94
61	Butyl benzoate	0 ± 0	0 ± 0	0 ± 0	0 ± 0	0 ± 0
62	Methyl salicylate	0 ± 0	0 ± 0	0 ± 0	0 ± 0	0 ± 0
63	2-tridecanone	0 ± 0	0 ± 0	0 ± 0	0 ± 0	0 ± 0
64	2-phenethyl acetate	0 ± 0	0 ± 0	0.31 ± 0.31	0.14 ± 0.15	0.3 ± 0.16
65	2-methoxy phenol	0 ± 0	0.09 ± 0.13	0.09 ± 0.11	0.12 ± 0.14	0.11 ± 0.14
66	Hexanoic acid	0 ± 0	4.37 ± 2.25	5.1 ± 2.12	6.77 ± 2.61	6.67 ± 0.47

Table S2. Relative amounts of volatile compounds in uninoculated and inoculated cacti. Volatile compounds (mean ± stdev) emitted from barrel, prickly pear, organ pipe and agria cacti, respectively.

	**Compound**	**Uninoculated Organ Pipe cactus**	**Week one Organ Pipe cactus rot**	**Week two Organ Pipe cactus rot**	**Week three Organ Pipe cactus rot**	**Week four Organ Pipe cactus rot**
67	Phenethyl propionate	0 ± 0	0 ± 0	0 ± 0	0 ± 0	0 ± 0
68	Isopentyl benzoate	0 ± 0	0 ± 0	0 ± 0	0 ± 0	0 ± 0
69	Phenethyl alcohol	0 ± 0	0.22 ± 0.05	0.05 ± 0.08	0 ± 0	0.16 ± 0.15
70	Creosol	0 ± 0	0.19 ± 0.03	0.21 ± 0.03	0.25 ± 0.04	0.16 ± 0.13
71	Phenol	0 ± 0	0.41 ± 0.64	0.46 ± 0.57	0.73 ± 1.02	0.81 ± 1.18
72	4-ethylguaiacol	0 ± 0	0 ± 0	0 ± 0	0 ± 0	0.04 ± 0.04
73	4-methyl phenol	0 ± 0	0 ± 0	0 ± 0	0 ± 0	0 ± 0
74	Octanoic acid	0 ± 0	0.89 ± 0.54	1.26 ± 0.13	1.43 ± 0.63	1.35 ± 0.19
75	4-ethyl phenol	0 ± 0	0 ± 0	0 ± 0	0 ± 0	0 ± 0
76	2-methoxy-4-propyl phenol	0 ± 0	0 ± 0	0 ± 0	0 ± 0	0 ± 0
77	4-vinylguaiacol	0 ± 0	0 ± 0	0 ± 0	0 ± 0	0 ± 0

Table S2. Relative amounts of volatile compounds in uninoculated and inoculated cacti. Volatile compounds (mean ± stdev) emitted from barrel, prickly pear, organ pipe and agria cacti, respectively.

	Compound	Week five Organ Pipe cactus rot	Week six Organ Pipe cactus rot	Week seven Organ Pipe cactus rot	Week eight Organ Pipe cactus rot	Week nine Organ Pipe cactus rot
1	Acetone	0.89 ± 1.54	2.93 ± 0.68	1.57 ± 1.47	2.72 ± 2.1	5.36 ± 1.05
2	2-butanone	0 ± 0	0 ± 0	0 ± 0	0 ± 0	0 ± 0
3	2-propanol	0 ± 0	0 ± 0	0 ± 0	0 ± 0	0 ± 0
4	Propyl acetate	3.55 ± 4.88	1.75 ± 3.03	0.53 ± 0.49	0 ± 0	0 ± 0
5	Isopropyl acetate	0 ± 0	0 ± 0	0 ± 0	0 ± 0	0 ± 0
6	Ethyl propionate	0 ± 0	0 ± 0	0 ± 0	0 ± 0	0 ± 0
7	Isopropyl propionate	0 ± 0	0 ± 0	0 ± 0	0 ± 0	0 ± 0
8	Propyl propionate	32.36 ± 16.06	27 ± 9.67	3.32 ± 3.12	0.15 ± 0.26	0 ± 0
9	6-methyl-2 Hepatanone	0 ± 0	0 ± 0	0 ± 0	0 ± 0	0 ± 0
10	Hexanal	0 ± 0	0 ± 0	0 ± 0	0 ± 0	0 ± 0
11	Ethyl butyrate	0 ± 0	0 ± 0	0 ± 0	0 ± 0	0 ± 0
12	Isopropyl isopentanoate	0 ± 0	0 ± 0	0 ± 0	0 ± 0	0 ± 0
13	Isopropyl pentanoate	0 ± 0	0 ± 0	0 ± 0	0 ± 0	0 ± 0
14	1-propanol	0 ± 0	0 ± 0	0 ± 0	0 ± 0	0 ± 0
15	Isobutanol	0 ± 0	0 ± 0	0 ± 0	0 ± 0	0 ± 0
16	Propyl butyrate	3.53 ± 1.73	2.91 ± 1.6	1.07 ± 0.93	0.06 ± 0.1	0 ± 0
17	Isopentyl acetate	0 ± 0	0 ± 0	0 ± 0	0 ± 0	0.15 ± 0.27
18	1-undecene	0 ± 0	0 ± 0	0 ± 0	0 ± 0	0 ± 0
19	1-dodecene	0 ± 0	0 ± 0	0 ± 0	0 ± 0	0 ± 0
20	Butyl propionate	1.14 ± 0.75	1.07 ± 1.21	0.23 ± 0.4	1.57 ± 2.17	1.36 ± 1.2
21	Pentanoic acid 1-methylpropyl ester	0 ± 0	0 ± 0	0 ± 0	0 ± 0	0 ± 0
22	Isopentyl propionate	0.25 ± 0.43	0.85 ± 1.48	0 ± 0	0 ± 0	5.09 ± 8.81
23	2-heptanone	0 ± 0	0 ± 0	0 ± 0	0 ± 0	0.29 ± 0.51
24	Butyl butyrate	0 ± 0	0 ± 0	0 ± 0	0 ± 0	0 ± 0
25	Isopentyl alcohol	0 ± 0	0 ± 0	0 ± 0	0.48 ± 0.83	0.35 ± 0.6
26	Hexanoic acid ethyl ester	0.37 ± 0.19	0.35 ± 0.36	0.17 ± 0.29	0 ± 0	0 ± 0
27	Hexanoic acid 1-methylethyl ester	0.28 ± 0.16	0.22 ± 0.3	0.15 ± 0.26	0 ± 0	0 ± 0
28	Isopropyl tiglate	0 ± 0	0 ± 0	0 ± 0	0 ± 0	0.13 ± 0.22

Table S2. Relative amounts of volatile compounds in uninoculated and inoculated cacti. Volatile compounds (mean ± stdev) emitted from barrel, prickly pear, organ pipe and agria cacti, respectively.

	Compound	Week five Organ Pipe cactus rot	Week six Organ Pipe cactus rot	Week seven Organ Pipe cactus rot	Week eight Organ Pipe cactus rot	Week nine Organ Pipe cactus rot
29	Isopentyl butyrate	0 ± 0	0 ± 0	0 ± 0	0 ± 0	0 ± 0
30	2-heptanol acetate	0 ± 0	0 ± 0	0.03 ± 0.05	0 ± 0	0 ± 0
31	3-octanone	0 ± 0	0 ± 0	0 ± 0	0 ± 0	0 ± 0
32	Hexyl acetate	0.58 ± 0.88	1.91 ± 3.3	0.24 ± 0.41	0 ± 0	0 ± 0
33	Acetoin	0 ± 0	0 ± 0	0 ± 0	0 ± 0	0 ± 0
34	n-propyl hexanoate	2.24 ± 0.82	1.1 ± 0.98	0 ± 0	0 ± 0	0 ± 0
35	Isobutyl tiglate	0 ± 0	0 ± 0	0 ± 0	0 ± 0	0 ± 0
36	Hexyl propionate	1.13 ± 1.95	1.25 ± 2.16	0.07 ± 0.11	0 ± 0	0 ± 0
37	2-methyl, 3-nonanol	0 ± 0	0 ± 0	0 ± 0	0 ± 0	0 ± 0
38	1-hexanol	0 ± 0	0 ± 0	0 ± 0	0 ± 0	0 ± 0
39	2-nonanone	0 ± 0	0 ± 0	0 ± 0	0 ± 0	0.72 ± 1.25
40	Durenol	0 ± 0	0 ± 0	0 ± 0	0 ± 0	0 ± 0
41	Butyl hexanoate	0 ± 0	0 ± 0	0 ± 0	0 ± 0	0 ± 0
42	Ethyl octanoate	0.16 ± 0.19	0.04 ± 0.08	0 ± 0	0 ± 0	0 ± 0
43	Isopentyl hexanoate	0 ± 0	0 ± 0	0 ± 0	0 ± 0	0 ± 0
44	2-octanol acetate	0 ± 0	0 ± 0	0 ± 0	0 ± 0	0 ± 0
45	N,N'-diethyl-1,3 benzenediamine	0 ± 0	0 ± 0	0 ± 0	0 ± 0	0 ± 0
46	2-nonanol	0 ± 0	0 ± 0	0 ± 0	0 ± 0	0 ± 0
47	Propyl octanoate	0.76 ± 0.41	0.13 ± 0.11	0 ± 0	0 ± 0	0 ± 0
48	Linalool	0 ± 0	0 ± 0	0 ± 0	0 ± 0	0 ± 0
49	Benzaldehyde	0 ± 0	0 ± 0	0 ± 0	0 ± 0	0 ± 0
50	Acetic acid	0 ± 0	0 ± 0	0 ± 0	0 ± 0	0 ± 0
51	Propionic acid	3.04 ± 1.57	1.17 ± 0.4	0.53 ± 0.49	0.59 ± 0.93	0 ± 0
52	n-propyl, 3-mercapto-propanoate	0 ± 0	0 ± 0	0 ± 0	0 ± 0	0 ± 0
53	Methyl benzoate	0 ± 0	0 ± 0	0 ± 0	0 ± 0	0.04 ± 0.06
54	Butyric acid	14.3 ± 7.31	3.69 ± 1.17	1.36 ± 0.57	1.58 ± 2.05	0.56 ± 0.97
55	Eugenol	0 ± 0	0 ± 0	0 ± 0	0 ± 0	0 ± 0
56	Isopropyl benzoate	0 ± 0	0 ± 0	0 ± 0	0 ± 0	0 ± 0
57	Acetophenone	0 ± 0	0 ± 0	0 ± 0	0 ± 0	0.04 ± 0.04
58	Ethyl benzoate	0 ± 0	0 ± 0	0 ± 0	0 ± 0	0 ± 0
59	Propyl benzoate	0 ± 0	0 ± 0	0 ± 0	0 ± 0	0 ± 0
60	Pentanoic acid	0.65 ± 1.12	0 ± 0	0 ± 0	0 ± 0	0 ± 0
61	Butyl benzoate	0 ± 0	0 ± 0	0 ± 0	0 ± 0	0 ± 0
62	Methyl salicylate	0 ± 0	0 ± 0	0 ± 0	0 ± 0	0 ± 0
63	2-tridecanone	0 ± 0	0 ± 0	0 ± 0	0 ± 0	0 ± 0
64	2-phenethyl acetate	0.07 ± 0.12	0.23 ± 0.4	0.2 ± 0.35	0 ± 0	0 ± 0
65	2-methoxy phenol	0.24 ± 0.37	0.2 ± 0.35	1.35 ± 1.69	0.79 ± 0.71	1.57 ± 1.79
66	Hexanoic acid	4.07 ± 0.71	1.09 ± 0.16	0.11 ± 0.2	0 ± 0	0 ± 0

Table S2. Relative amounts of volatile compounds in uninoculated and inoculated cacti. Volatile compounds (mean ± stdev) emitted from barrel, prickly pear, organ pipe and agria cacti, respectively.

	Compound	Week five Organ Pipe cactus rot	Week six Organ Pipe cactus rot	Week seven Organ Pipe cactus rot	Week eight Organ Pipe cactus rot	Week nine Organ Pipe cactus rot
67	Phenethyl propionate	0 ± 0	0.49 ± 0.27	0 ± 0	0 ± 0	0 ± 0
68	Isopentyl benzoate	0 ± 0	0 ± 0	0 ± 0	0 ± 0	0 ± 0
69	Phenethyl alcohol	0.22 ± 0.19	0.18 ± 0.15	0.24 ± 0.07	0.21 ± 0.19	0.68 ± 0.27
70	Creosol	0.16 ± 0.14	0.13 ± 0.11	0.11 ± 0.11	0.1 ± 0.08	0.25 ± 0.23
71	Phenol	1.11 ± 1.81	1.09 ± 1.72	2.55 ± 1.34	2.27 ± 1.38	1.56 ± 1.11
72	4-ethylguaiacol	0.05 ± 0.04	0.02 ± 0.04	0.03 ± 0.03	0.03 ± 0.02	0.07 ± 0.06
73	4-methyl phenol	0 ± 0	0 ± 0	0 ± 0	0 ± 0	0 ± 0
74	Octanoic acid	0.71 ± 0.17	0 ± 0	0 ± 0	0 ± 0	0 ± 0
75	4-ethyl phenol	0 ± 0	0 ± 0	0 ± 0	0 ± 0	0 ± 0
76	2-methoxy-4-propyl phenol	0 ± 0	0 ± 0	0 ± 0	0 ± 0	0 ± 0
77	4-vinylguaiacol	0 ± 0	0 ± 0	0 ± 0	0 ± 0	0 ± 0

Table S2. Relative amounts of volatile compounds in uninoculated and inoculated cacti. Volatile compounds (mean ± stdev) emitted from barrel, prickly pear, organ pipe and agria cacti, respectively.

	Compound	Uninoculated Agria cactus	Week one Agria cactus rot	Week two Agria cactus rot	Week three Agria cactus rot	Week four Agria cactus rot
1	Acetone	0 ± 0	0 ± 0	0 ± 0	0 ± 0	0 ± 0
2	2-butanone	0 ± 0	0 ± 0	0 ± 0	0 ± 0	0 ± 0
3	2-propanol	0 ± 0	2.78 ± 1.06	1.57 ± 1.62	3.62 ± 0.61	2.21 ± 2.14
4	Propyl acetate	0 ± 0	0.37 ± 0.65	1.46 ± 1.57	2.83 ± 0.89	2.4 ± 1.9
5	Isopropyl acetate	0 ± 0	0 ± 0	0 ± 0	0 ± 0	0 ± 0
6	Ethyl propionate	0 ± 0	0 ± 0	0 ± 0	0 ± 0	0 ± 0
7	Isopropyl propionate	0 ± 0	0 ± 0	0 ± 0	0 ± 0	0 ± 0
8	Propyl propionate	0 ± 0	0 ± 0	0 ± 0	0 ± 0	0 ± 0
9	6-methyl-2 Hepatanone	1.33 ± 0.18	0.04 ± 0.03	0.38 ± 0.44	0.8 ± 0.55	1.01 ± 1.01
10	Hexanal	0.78 ± 0.1	0 ± 0	0 ± 0	0 ± 0	0 ± 0
11	Ethyl butyrate	0 ± 0	0.35 ± 0.6	0.94 ± 0.84	1.28 ± 1.28	1 ± 0.8
12	Isopropyl isopentanoate	0 ± 0	0 ± 0	0 ± 0	0 ± 0	0 ± 0
13	Isopropyl pentanoate	0 ± 0	0 ± 0	0 ± 0	0 ± 0	0 ± 0
14	1-propanol	0 ± 0	2.8 ± 4.85	1.24 ± 2.15	4.74 ± 2.29	5.05 ± 3.62
15	Isobutanol	0 ± 0	0 ± 0	0 ± 0	0 ± 0	0 ± 0
16	Propyl butyrate	0 ± 0	4.73 ± 4.27	6.56 ± 6.89	20.16 ± 2.27	10.55 ± 7.53
17	Isopentyl acetate	0 ± 0	0 ± 0	0 ± 0	0 ± 0	0 ± 0
18	1-undecene	0 ± 0	0 ± 0	0 ± 0	0 ± 0	0 ± 0
19	1-dodecene	0 ± 0	0 ± 0	0 ± 0	0 ± 0	0 ± 0
20	Butyl propionate	0 ± 0	0 ± 0	0.11 ± 0.19	0 ± 0	0 ± 0
21	Pentanoic acid 1-methylpropyl ester	0 ± 0	0 ± 0	0 ± 0	0 ± 0	0 ± 0
22	Isopentyl propionate	0 ± 0	0 ± 0	0 ± 0	0 ± 0	0 ± 0
23	2-heptanone	0 ± 0	0 ± 0	0 ± 0	0 ± 0	0 ± 0
24	Butyl butyrate	0 ± 0	2.93 ± 2.08	2.2 ± 3.81	4.92 ± 3.51	2.99 ± 4.61
25	Isopentyl alcohol	0 ± 0	0 ± 0	0 ± 0	0 ± 0	0 ± 0
26	Hexanoic acid ethyl ester	0 ± 0	0 ± 0	0.11 ± 0.19	0 ± 0	0.05 ± 0.09
27	Hexanoic acid 1-methylethyl ester	0 ± 0	0 ± 0	0 ± 0	0 ± 0	0 ± 0
28	Isopropyl tiglate	0 ± 0	0 ± 0	0 ± 0	0 ± 0	0 ± 0

Table S2. Relative amounts of volatile compounds in uninoculated and inoculated cacti. Volatile compounds (mean ± stdev) emitted from barrel, prickly pear, organ pipe and agria cacti, respectively.

	Compound	Uninoculated Agria cactus	Week one Agria cactus rot	Week two Agria cactus rot	Week three Agria cactus rot	Week four Agria cactus rot
29	Isopentyl butyrate	0 ± 0	0.17 ± 0.14	0.11 ± 0.19	0.77 ± 0.37	0.62 ± 0.62
30	2-heptanol acetate	0 ± 0	0 ± 0	0 ± 0	0 ± 0	0 ± 0
31	3-octanone	0 ± 0	0 ± 0	0 ± 0	0 ± 0	0 ± 0
32	Hexyl acetate	0 ± 0	0 ± 0	0 ± 0	0 ± 0	0 ± 0
33	Acetoin	0 ± 0	0 ± 0	0 ± 0	0 ± 0	0 ± 0
34	n-propyl hexanoate	0 ± 0	0.43 ± 0.07	0.68 ± 0.77	2 ± 1.59	0.97 ± 1.14
35	Isobutyl tiglate	0 ± 0	0 ± 0	0 ± 0	0 ± 0	0 ± 0
36	Hexyl propionate	0 ± 0	0 ± 0	0 ± 0	0 ± 0	0 ± 0
37	2-methyl, 3-nonanol	0 ± 0	0 ± 0	0 ± 0	0 ± 0	0 ± 0
38	1-hexanol	0 ± 0	0 ± 0	0.04 ± 0.08	0 ± 0	0 ± 0
39	2-nonanone	0 ± 0	0 ± 0	0 ± 0	0 ± 0	0 ± 0
40	Durenol	0 ± 0	0 ± 0	0 ± 0	0 ± 0	0 ± 0
41	Butyl hexanoate	0 ± 0	0 ± 0	0 ± 0	0 ± 0	0 ± 0
42	Ethyl octanoate	0 ± 0	0.05 ± 0.09	0.31 ± 0.3	0.34 ± 0.09	0.26 ± 0.19
43	Isopentyl hexanoate	0 ± 0	0 ± 0	0 ± 0	0 ± 0	0 ± 0
44	2-octanol acetate	0 ± 0	0 ± 0	0 ± 0	0 ± 0	0 ± 0
45	N,N'-diethyl-1,3 benzenediamine	0 ± 0	0 ± 0	0 ± 0	0 ± 0	0 ± 0
46	2-nonanol	0 ± 0	0 ± 0	0 ± 0	0 ± 0	0 ± 0
47	Propyl octanoate	0 ± 0	0.08 ± 0.15	0.15 ± 0.26	0.41 ± 0.37	0.51 ± 0.37
48	Linalool	0 ± 0	0 ± 0	0 ± 0	0 ± 0	0 ± 0
49	Benzaldehyde	0.03 ± 0.02	0 ± 0	0 ± 0	0 ± 0	0 ± 0
50	Acetic acid	0 ± 0	2.77 ± 3.44	3.05 ± 3.1	3.22 ± 0.12	1.95 ± 1.18
51	Propionic acid	0 ± 0	0.82 ± 0.45	0.23 ± 0.39	0.57 ± 0.98	0 ± 0
52	n-propyl, 3-mercapto-propanoate	0 ± 0	0 ± 0	0 ± 0	0 ± 0	0 ± 0
53	Methyl benzoate	0 ± 0	0 ± 0	0 ± 0	0 ± 0	0 ± 0
54	Butyric acid	0 ± 0	43.38 ± 20.57	29.22 ± 20.1	37.66 ± 12.33	21.24 ± 16.08
55	Eugenol	0 ± 0	0 ± 0	0 ± 0	0 ± 0	0 ± 0
56	Isopropyl benzoate	0 ± 0	0 ± 0	7.54 ± 6.68	11.94 ± 0.71	7.54 ± 6.57
57	Acetophenone	0 ± 0	2.69 ± 4.66	0 ± 0	0 ± 0	0 ± 0
58	Ethyl benzoate	0 ± 0	6.45 ± 1.45	7.76 ± 2.54	7.48 ± 1.75	6.09 ± 2.31
59	Propyl benzoate	0 ± 0	8.99 ± 0.43	14.58 ± 4.09	13.24 ± 3.99	8.45 ± 5.98
60	Pentanoic acid	0 ± 0	1.17 ± 0.54	1.41 ± 1.32	0.89 ± 0.86	0 ± 0
61	Butyl benzoate	0 ± 0	0.47 ± 0.65	0.69 ± 0.19	1.04 ± 0.72	1.82 ± 1.81
62	Methyl salicylate	0 ± 0	0 ± 0	0 ± 0	0 ± 0	0 ± 0
63	2-tridecanone	0 ± 0	0 ± 0	0 ± 0	0 ± 0	0 ± 0
64	2-phenethyl acetate	0 ± 0	0 ± 0	0 ± 0	0 ± 0	0 ± 0
65	2-methoxy phenol	0 ± 0	0.32 ± 0.28	0 ± 0	0 ± 0	0 ± 0
66	Hexanoic acid	0 ± 0	0.81 ± 0.24	1.54 ± 1.9	3.24 ± 2.83	1.78 ± 1.15

Table S2. Relative amounts of volatile compounds in uninoculated and inoculated cacti. Volatile compounds (mean ± stdev) emitted from barrel, prickly pear, organ pipe and agria cacti, respectively.

	Compound	Uninoculated Agria cactus	Week one Agria cactus rot	Week two Agria cactus rot	Week three Agria cactus rot	Week four Agria cactus rot
67	Phenethyl propionate	0 ± 0	0 ± 0	0 ± 0	0 ± 0	0 ± 0
68	Isopentyl benzoate	0 ± 0	0 ± 0	0 ± 0	0 ± 0	0 ± 0
69	Phenethyl alcohol	0 ± 0	0.43 ± 0.11	0.46 ± 0.05	0.35 ± 0.08	0.34 ± 0.23
70	Creosol	0 ± 0	0 ± 0	0.16 ± 0.27	0.11 ± 0.19	0.01 ± 0.02
71	Phenol	0 ± 0	1.39 ± 1.15	1.74 ± 0.76	1.71 ± 0.78	1.29 ± 1.15
72	4-ethylguaiacol	0 ± 0	0 ± 0	0 ± 0	0 ± 0	0 ± 0
73	4-methyl phenol	0 ± 0	0 ± 0	0 ± 0	0 ± 0	0 ± 0
74	Octanoic acid	0 ± 0	0.32 ± 0.27	0.61 ± 0.26	0.48 ± 0.04	0.42 ± 0.38
75	4-ethyl phenol	0 ± 0	0 ± 0	0 ± 0	0 ± 0	0 ± 0
76	2-methoxy-4-propyl phenol	0 ± 0	0 ± 0	0 ± 0	0 ± 0	0 ± 0
77	4-vinylguaiacol	0 ± 0	0 ± 0	0 ± 0	0 ± 0	0 ± 0

Table S2. Relative amounts of volatile compounds in uninoculated and inoculated cacti. Volatile compounds (mean ± stdev) emitted from barrel, prickly pear, organ pipe and agria cacti, respectively.

	Compound	Week five Agria cactus rot	Week six Agria cactus rot	Week seven Agria cactus rot	Week eight Agria cactus rot	Week nine Agria cactus rot
1	Acetone	0 ± 0	0 ± 0	0 ± 0	0 ± 0	0 ± 0
2	2-butanone	0 ± 0	0 ± 0	0 ± 0	0 ± 0	0 ± 0
3	2-propanol	0.55 ± 0.96	0.48 ± 0.84	0.69 ± 0.99	1.55 ± 1.68	2.19 ± 0.28
4	Propyl acetate	1.22 ± 0.53	1.07 ± 0.78	0.5 ± 0.74	0.86 ± 0.75	2.16 ± 0.36
5	Isopropyl acetate	0 ± 0	0 ± 0	0 ± 0	0 ± 0	0 ± 0
6	Ethyl propionate	0 ± 0	0 ± 0	0 ± 0	0 ± 0	0 ± 0
7	Isopropyl propionate	0 ± 0	0 ± 0	0 ± 0	0 ± 0	0 ± 0
8	Propyl propionate	0 ± 0	0 ± 0	0 ± 0	0 ± 0	0 ± 0
9	6-methyl-2 Hepatanone	1.15 ± 0.61	0.81 ± 0.58	0.95 ± 1.09	1.04 ± 0.91	2.36 ± 0.67
10	Hexanal	0 ± 0	0 ± 0	0 ± 0	0 ± 0	0 ± 0
11	Ethyl butyrate	1.03 ± 0.44	0.78 ± 0.61	0.32 ± 0.33	0.55 ± 0.52	1.16 ± 0.36
12	Isopropyl isopentanoate	0 ± 0	0 ± 0	0 ± 0	0 ± 0	0 ± 0
13	Isopropyl pentanoate	0 ± 0	0 ± 0	0 ± 0	0 ± 0	0 ± 0
14	1-propanol	2.29 ± 2.28	3.45 ± 2.47	1.86 ± 2.46	1.61 ± 2	5.47 ± 0.83
15	Isobutanol	0 ± 0	0 ± 0	0 ± 0	0 ± 0	0 ± 0
16	Propyl butyrate	11.94 ± 2.54	6.1 ± 4.15	5.28 ± 5.54	7.75 ± 6.24	14.03 ± 2.98
17	Isopentyl acetate	0 ± 0	0 ± 0	0 ± 0	0 ± 0	0 ± 0
18	1-undecene	0 ± 0	0 ± 0	0 ± 0	0 ± 0	0 ± 0
19	1-dodecene	0 ± 0	0 ± 0	0 ± 0	0 ± 0	0 ± 0
20	Butyl propionate	0 ± 0	0 ± 0	0 ± 0	0.3 ± 0.29	0 ± 0
21	Pentanoic acid 1-methylpropyl ester	0 ± 0	0 ± 0	0 ± 0	0 ± 0	0 ± 0
22	Isopentyl propionate	0 ± 0	0 ± 0	0 ± 0	0 ± 0	0 ± 0
23	2-heptanone	0 ± 0	0 ± 0	0 ± 0	0 ± 0	0 ± 0
24	Butyl butyrate	0.9 ± 1.56	1.44 ± 1.92	2.04 ± 3.3	1.74 ± 2.82	3.55 ± 3.71
25	Isopentyl alcohol	0 ± 0	0 ± 0	0 ± 0	0 ± 0	0 ± 0
26	Hexanoic acid ethyl ester	0.43 ± 0.75	0.56 ± 0.96	0.24 ± 0.42	0.37 ± 0.6	0.52 ± 0.91
27	Hexanoic acid 1-methylethyl ester	0 ± 0	0 ± 0	0 ± 0	0 ± 0	0 ± 0
28	Isopropyl tiglate	0 ± 0	0 ± 0	0 ± 0	0 ± 0	0 ± 0

Table S2. Relative amounts of volatile compounds in uninoculated and inoculated cacti. Volatile compounds (mean ± stdev) emitted from barrel, prickly pear, organ pipe and agria cacti, respectively.

	Compound	Week five Agria cactus rot	Week six Agria cactus rot	Week seven Agria cactus rot	Week eight Agria cactus rot	Week nine Agria cactus rot
29	Isopentyl butyrate	0 ± 0	0.32 ± 0.55	0.23 ± 0.26	0 ± 0	0 ± 0
30	2-heptanol acetate	0 ± 0	0 ± 0	0 ± 0	0 ± 0	0 ± 0
31	3-octanone	0 ± 0	0 ± 0	0 ± 0	0 ± 0	0 ± 0
32	Hexyl acetate	0 ± 0	0 ± 0	0 ± 0	0 ± 0	0 ± 0
33	Acetoin	0 ± 0	0 ± 0	0 ± 0	0 ± 0	0 ± 0
34	n-propyl hexanoate	2.16 ± 3.21	0.79 ± 1.32	1.54 ± 1.97	1.64 ± 2.21	3.6 ± 2.93
35	Isobutyl tiglate	0 ± 0	0 ± 0	0 ± 0	0 ± 0	0 ± 0
36	Hexyl propionate	0 ± 0	0 ± 0	0 ± 0	0.66 ± 1.14	0 ± 0
37	2-methyl, 3-nonanol	0 ± 0	0 ± 0	0 ± 0	0 ± 0	0 ± 0
38	1-hexanol	0 ± 0	0 ± 0	0.04 ± 0.07	0 ± 0	0 ± 0
39	2-nonanone	0 ± 0	0 ± 0	0 ± 0	0 ± 0	0 ± 0
40	Durenol	0 ± 0	0 ± 0	0 ± 0	0 ± 0	0 ± 0
41	Butyl hexanoate	0 ± 0	0 ± 0	0.04 ± 0.07	0 ± 0	0 ± 0
42	Ethyl octanoate	0.29 ± 0.04	0.14 ± 0.13	0.24 ± 0.21	0.14 ± 0.12	0.24 ± 0.12
43	Isopentyl hexanoate	0 ± 0	0 ± 0	0 ± 0	0 ± 0	0 ± 0
44	2-octanol acetate	0 ± 0	0 ± 0	0 ± 0	0 ± 0	0 ± 0
45	N,N'-diethyl-1,3 benzenediamine	0 ± 0	0 ± 0	0 ± 0	0 ± 0	0 ± 0
46	2-nonanol	0 ± 0	0 ± 0	0 ± 0	0 ± 0	0 ± 0
47	Propyl octanoate	0.36 ± 0.32	0.02 ± 0.04	0.18 ± 0.25	0 ± 0	0.49 ± 0.42
48	Linalool	0 ± 0	0 ± 0	0 ± 0	0 ± 0	0 ± 0
49	Benzaldehyde	0 ± 0	0.01 ± 0.01	0 ± 0	0 ± 0	0 ± 0
50	Acetic acid	3.66 ± 2.84	3.46 ± 3.16	1.77 ± 1.56	2.05 ± 1.65	3.83 ± 2.56
51	Propionic acid	0 ± 0	0 ± 0	0.47 ± 0.81	0.47 ± 0.81	1.53 ± 0.65
52	n-propyl, 3-mercapto-propanoate	0 ± 0	0 ± 0	0 ± 0	0 ± 0	0 ± 0
53	Methyl benzoate	0 ± 0	0 ± 0	0.1 ± 0.09	0 ± 0	0 ± 0
54	Butyric acid	25.06 ± 8.81	22.86 ± 16.34	17.74 ± 16.94	16.3 ± 13.41	28.05 ± 6.31
55	Eugenol	0 ± 0	0 ± 0	0 ± 0	0 ± 0	0 ± 0
56	Isopropyl benzoate	8.16 ± 0.44	7.33 ± 3.4	4.73 ± 4.1	5.06 ± 3.06	10.09 ± 1.04
57	Acetophenone	0 ± 0	0 ± 0	0 ± 0	0 ± 0	0 ± 0
58	Ethyl benzoate	6.24 ± 1.08	4.39 ± 3.84	3.38 ± 3.01	3.35 ± 2.91	7.26 ± 1.3
59	Propyl benzoate	9.42 ± 3.16	5.57 ± 4.24	5.17 ± 3.78	4.56 ± 3.45	9.37 ± 2.85
60	Pentanoic acid	1.79 ± 1.59	1.01 ± 1.64	0.73 ± 0.7	1.1 ± 0.91	2.19 ± 0.84
61	Butyl benzoate	0.73 ± 0.79	0 ± 0	0.3 ± 0.53	0 ± 0	0 ± 0
62	Methyl salicylate	0 ± 0	0 ± 0	0 ± 0	0 ± 0	0 ± 0
63	2-tridecanone	0 ± 0	0 ± 0	0 ± 0	0 ± 0	0 ± 0
64	2-phenethyl acetate	0 ± 0	0 ± 0	0 ± 0	0 ± 0	0 ± 0
65	2-methoxy phenol	0 ± 0	0 ± 0	0.17 ± 0.15	0.36 ± 0.44	0.98 ± 0.86
66	Hexanoic acid	3.69 ± 3.67	1.06 ± 1.52	1.79 ± 2.31	1.71 ± 2.29	3.43 ± 3.56

Table S2. Relative amounts of volatile compounds in uninoculated and inoculated cacti. Volatile compounds (mean ± stdev) emitted from barrel, prickly pear, organ pipe and agria cacti, respectively.

	**Compound**	**Week five Agria cactus rot**	**Week six Agria cactus rot**	**Week seven Agria cactus rot**	**Week eight Agria cactus rot**	**Week nine Agria cactus rot**
67	Phenethyl propionate	0 ± 0	0 ± 0	0 ± 0	0 ± 0	0 ± 0
68	Isopentyl benzoate	0 ± 0	0 ± 0	0 ± 0	0 ± 0	0 ± 0
69	Phenethyl alcohol	0.36 ± 0.05	0.26 ± 0.23	0.19 ± 0.17	0.22 ± 0.14	0.56 ± 0.1
70	Creosol	0 ± 0	0.06 ± 0.1	0.11 ± 0.1	0.09 ± 0.07	0.26 ± 0.04
71	Phenol	1.22 ± 0.51	0.74 ± 0.45	0.78 ± 0.77	0.72 ± 0.64	1.29 ± 0.59
72	4-ethylguaiacol	0 ± 0	0 ± 0	0 ± 0	0 ± 0	0 ± 0
73	4-methyl phenol	0 ± 0	0 ± 0	0 ± 0	0 ± 0	0 ± 0
74	Octanoic acid	0.49 ± 0.14	0.23 ± 0.2	0 ± 0	0 ± 0	0 ± 0
75	4-ethyl phenol	0 ± 0	0 ± 0	0 ± 0	0 ± 0	0 ± 0
76	2-methoxy-4-propyl phenol	0 ± 0	0 ± 0	0 ± 0	0 ± 0	0 ± 0
77	4-vinylguaiacol	0 ± 0	0 ± 0	0 ± 0	0 ± 0	0 ± 0
