# Supplementary material for: Divergence in Olfactory Host Plant Preference in D. mojavensis in Response to Cactus Host Use
Source: PLoS One. 2013 Jul 25;8(7):e70027. doi: 10.1371/journal.pone.0070027 (PMC3723661; doi:10.1371/journal.pone.0070027)
Supplement: Table S3 — Principal component values for volatile compounds in the four host cacti. Eigenvectors with highest scores are indicated in bold. The compounds which were present only once across all four cacti were excluded from the PCA. (PDF) [file pone.0070027.s004.pdf]

Table S3. Principal component values for volatile compounds in the four host cacti. Eigenvectors with highest scores are indicated in bold. The compounds which were present only once across all four cacti were excluded from the PCA.

		PC 1	PC 2	PC 3	PC 4
	**Eigenvalue**	11.17	6.14	5.64	4.35
	**Percent variation**	16.67	9.17	8.42	6.5
	**Eigenvectors**				
1	Acetone	**-0.15226**	-0.00047	-0.00702	-0.03604
2	2-butanone	-0.08536	-0.01274	-0.09704	**-0.13253**
3	2 propanol	0.18833	0.12607	0.07016	-0.03027
4	Propyl acetate	0.054	-0.11443	**0.14793**	0.16297
5	Isopropyl acetate	-0.06172	0.01305	0.0724	0.0549
7	Isopropyl propionate	-0.09111	0.04925	0.09293	0.06382
8	Propyl propionate	0.0741	**-0.30041**	0.10396	0.16899
9	2 Hepatanone-6-methyl	0.16395	0.19732	0.05887	-0.08688
10	Hexanal	0	0	0	0
11	Ethyl butyrate	0.11608	0.12472	-0.22076	0.17835
14	1 propanol	0.18336	**0.20222**	0.06682	-0.07062
15	Isobutanol	-0.03763	0.00385	-0.06987	0.01991
16	Propyl butyrate	**0.23966**	0.14197	0.10237	-0.03567
17	Isopentyl acetate	-0.11877	0.04524	0.09879	0.07218
18	1-undecene	-0.04885	0.0716	0.06029	0.12015
19	1-dodecene	0	0	0	0
20	Butyl propionate	0.04648	**-0.24057**	0.07452	0.07772
22	Isopentyl propionate	-0.10498	0.03614	0.07952	0.05025
23	2-heptanone	-0.03494	0.01715	0.02463	0.00376
24	Butyl butyrate	0.13302	0.18268	-0.17907	0.12966
25	Isopentyl alcohol	-0.11821	0.06262	-0.03083	0.04142
26	Hexanoic acid ethyl ester	0.13181	-0.01322	-0.16152	**0.22981**
27	Hexanoic acid 1-methylethyl ester	-0.00323	-0.06487	-0.16539	0.00763
28	Isopropyl tiglate	-0.04036	-0.02147	-0.06751	**-0.09703**
29	Isopentyl butyrate	0.06218	0.09121	**-0.28514**	**0.24756**
30	2-heptanol acetate	-0.06436	0.01829	0.06829	0.10127
31	3-octanone	-0.05919	0.02588	-0.01994	-0.01978
32	Hexyl acetate	-0.01417	-0.12534	0.07539	0.18671
33	Acetoin	-0.0938	0.07482	-0.0326	0.10478
34	n-propyl hexanoate	**0.19887**	-0.09242	0.09302	0.11495
36	Hexyl propionate	0.05114	-0.17666	0.06625	0.09265

38	1-hexanol	-0.02394	0.09305	0.00829	0.16031
39	2-nonanone	-0.13418	0.094	0.09093	0.12672
40	Durenol	-0.11164	0.10302	0.07656	0.14628
41	Butyl hexanoate	0.04138	0.04846	**-0.28867**	**0.26583**
42	Ethyl octanoate	**0.22212**	-0.04584	**0.12897**	0.06963
43	Isopentyl hexanoate	0.04143	0.04918	**-0.29398**	**0.27116**
45	N,N'-diethyl-1,3 benzenediamine	**-0.14839**	0.113	0.10076	0.13669
46	2-nonanol	-0.09605	0.10135	0.10516	0.17092
47	Propyl octanoate	0.1392	**-0.19144**	0.1113	0.11482
48	Linalool	-0.03716	-0.03815	**-0.26168**	**-0.09355**
49	Benzaldehyde	-0.06001	0.04485	0.02075	0.04721
50	Acetic acid	0.18757	**0.19907**	0.06044	-0.07669
51	Propionic acid	0.11916	**-0.23402**	0.10151	0.12677
53	Methyl benzoate	-0.13968	0.09016	0.05299	0.10007
54	Butyric acid	**0.24939**	0.06513	-0.03072	0.13701
55	Eugenol	-0.05883	0.01968	-0.04557	-0.01882
56	Isopropyl benzoate	0.19296	**0.2521**	0.01073	-0.02371
57	Acetophenone	0.00714	0.0121	-0.00345	-0.02824
58	Ethyl benzoate	0.15967	**0.27103**	0.07798	0.00393
59	Propyl benzoate	0.18452	**0.25143**	0.09324	-0.06709
60	Pentanoic acid	0.15397	0.02081	0.07624	0.00803
61	Butyl benzoate	0.09834	0.18151	0.05954	-0.05902
62	Methyl salicylate	-0.02469	0.03906	**-0.2849**	0.17138
63	2-tridecanone	**-0.15445**	0.10034	0.12574	0.14712
64	2-phenethyl acetate	-0.08434	0.01772	-0.04639	0.04839
65	2-methoxy Phenol	**-0.18379**	0.13963	**0.13795**	0.18359
66	Hexanoic acid	**0.19454**	-0.11227	-0.04193	**0.24369**
67	Phenethyl propionate	-0.11678	0.03555	0.06729	0.04815
68	Isopentyl benzoate	-0.08724	0.06157	-0.00938	0.03518
69	Phenethyl alcohol	-0.10033	0.04334	-0.13636	**-0.11149**
70	Creosol	-0.08706	0.03817	**0.13225**	0.10235
71	Phenol	-0.05028	0.17009	**0.14737**	0.07749
72	4-ethylguaiacol	-0.10716	0.08344	0.04778	0.106
73	4-methyl phenol	-0.04319	-0.04833	-0.14834	**-0.16518**
74	Octanoic acid	0.1627	**-0.18193**	0.11428	0.12793
75	4-ethyl phenol	-0.08213	-0.01259	-0.25463	-0.07991
76	2-methoxy-4-propyl phenol	-0.12883	0.09395	0.07663	0.12175
77	4-vinylguaiacol	**-0.15658**	0.10736	0.08025	0.15366
