# Supplementary material for: Correction: Divergence in Olfactory Host Plant Preference in D. mojavensis in Response to Cactus Host Use
Source: PLoS One. 2014 Feb 18;9(2):e90050. doi: 10.1371/journal.pone.0090050 (PMC3928447; doi:10.1371/journal.pone.0090050)
Supplement: Figure S1 — Analysis of host plant volatile composition with cactus rot stage. Cacti were either uninoculated (NI) or inoculated and fermented for one to nine weeks. Peak numbers correspond to the list of volatiles. (A–D) Typical gas chromatograms of barrel, prickly pear, organ pipe and agria headspace (respectively) from uninoculated or representative fermented samples (weeks 1, 5, and 9). (PDF) [file pone.0090050.s001.pdf]

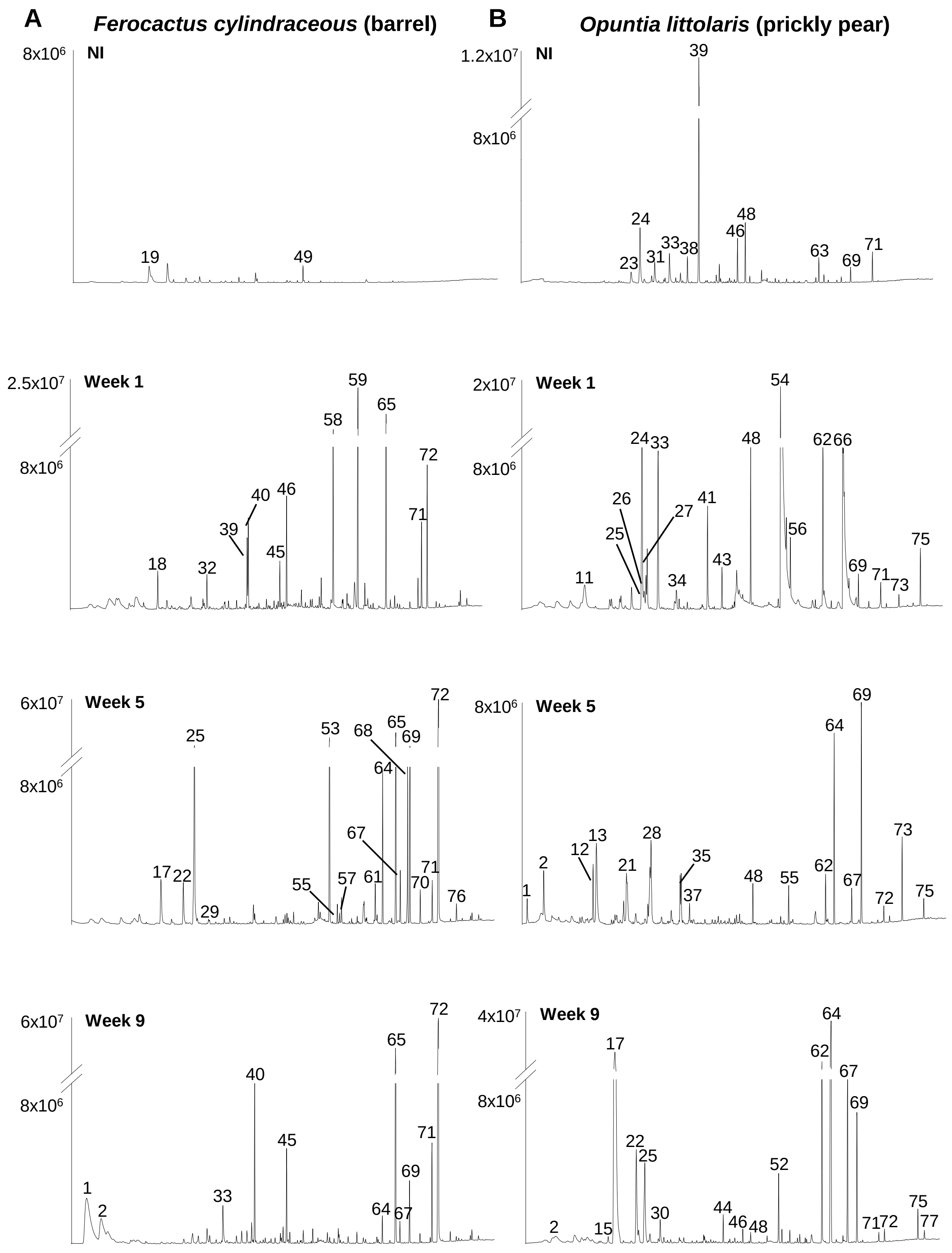
A
Ferocactus cylindraceous (barrel)
B
Opuntia littolaris (prickly pear)
8x10⁶ NI
19 49
1.2x10⁷ NI
8x10⁶
39 24 48 46 33 23 31 38 63 71 69
2.5x10⁷ Week 1
8x10⁶
59 58 65 72 40 46 39 71 45 18 32
2x10⁷ Week 1
8x10⁶
54 24 33 48 62 66 26 27 41 25 56 75 11 43 34 69 71 73
6x10⁷ Week 5
8x10⁶
72 25 53 68 65 69 64 67 17 22 55 57 61 71 70 76 29
8x10⁶ Week 5
69 64 73 13 12 28 35 2 21 62 1 48 55 67 37 72 75
6x10⁷ Week 9
8x10⁶
72 65 40 45 71 1 69 33 2 64 67
4x10⁷ Week 9
8x10⁶
64 17 62 67 69 22 25 52 75 44 2 30 46 48 15 71 72 77

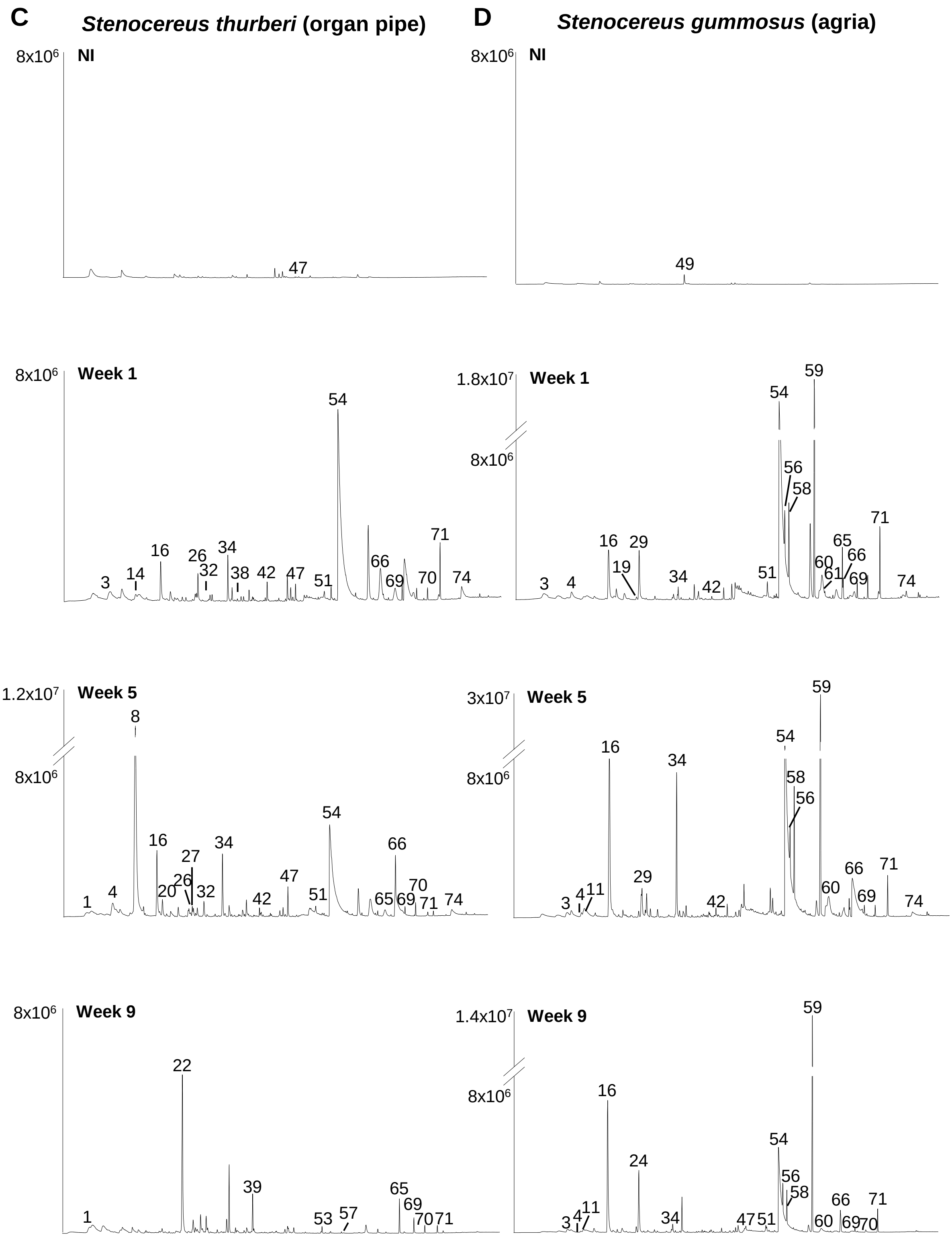
C
Stenocereus thurberi (organ pipe)
8x10⁶ NI
47
8x10⁶ Week 1
3 14 16 26 32 34 38 42 47 51 54 66 69 70 71 74
1.2x10⁷ Week 5
8x10⁶
1 4 8 16 20 26 27 32 34 42 47 51 54 65 66 69 70 71 74
8x10⁶ Week 9
1 22 39 53 57 65 69 70 71
D
Stenocereus gummosus (agria)
8x10⁶ NI
49
1.8x10⁷ Week 1
8x10⁶
3 4 16 19 29 34 42 51 54 56 58 59 60 61 65 66 69 71 74
3x10⁷ Week 5
8x10⁶
3 4 11 16 29 34 42 54 56 58 59 60 66 69 71 74
1.4x10⁷ Week 9
8x10⁶
3 4 11 16 24 34 47 51 54 56 58 59 60 66 69 70 71
